# Anti‐Environmental Aging Passive Daytime Radiative Cooling

**DOI:** 10.1002/advs.202305664

**Published:** 2023-12-26

**Authors:** Jianing Song, Qingchen Shen, Huijuan Shao, Xu Deng

**Affiliations:** ^1^ Institute of Fundamental and Frontier Sciences University of Electronic Science and Technology of China Chengdu 610054 China; ^2^ Bio‐inspired Photonics Group Yusuf Hamied Department of Chemistry University of Cambridge Lensfield Road Cambridge CB2 1EW UK

**Keywords:** anti‐contamination, anti‐environmental aging, passive daytime radiative cooling, photothermal durability, surface wettability

## Abstract

Passive daytime radiative cooling technology presents a sustainable solution for combating global warming and accompanying extreme weather, with great potential for diverse applications. The key characteristics of this cooling technology are the ability to reflect most sunlight and radiate heat through the atmospheric transparency window. However, the required high solar reflectance is easily affected by environmental aging, rendering the cooling ineffective. In recent years, significant advancements have been made in understanding the failure mechanisms, design strategies, and manufacturing technologies of daytime radiative cooling. Herein, a critical review on anti‐environmental aging passive daytime radiative cooling with the goal of advancing their commercial applications is presented. It is first introduced the optical mechanisms and optimization principles of radiative cooling, which serve as a basis for further endowing environmental durability. Then the environmental aging conditions of passive daytime radiative cooling, mainly focusing on UV exposure, thermal aging, surface contamination and chemical corrosion are discussed. Furthermore, the developments of anti‐environmental aging passive daytime radiative cooling materials, including design strategies, fabrication techniques, structures, and performances, are reviewed and classified for the first time. Last but not the least, the remaining open challenges and the insights are presented for the further promotion of the commercialization progress.

## Graphical abstract

In this paper, the passive daytime radiative cooling against environmental aging is reviewed with the goal of advancing their commercial applications. This paper introduces the optical mechanisms and optimization principles of PDRC, discusses the environmental aging conditions of PDRC, and focuses on developments of anti‐environmental aging passive daytime radiative cooling materials, including design strategies, fabrication techniques, structures and performances.

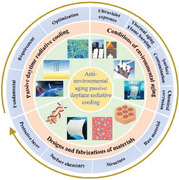

## 1 Introduction

Since the onset of the industrial revolution, the continuous discharge of heat‐absorbing greenhouse gases, such as carbon dioxide, into the atmosphere by human activities has led to a gradual increase of the global average temperature.^[^ ^1^ ^]^ This has resulted in a myriad of problems, including extreme heat waves,^[^ ^2^ ^]^ melting glaciers,^[^ ^3^ ^]^ floods^[^ ^4^ ^]^ and rising biological mortality^[^ ^5^ ^]^ which have drawn the attention of countries worldwide. Despite the Paris Agreement providing a new framework for global cooperation to address climate change, achieving the target of limiting global warming to 1.5 °C remains a daunting challenge, as greenhouse gas emissions are still soaring.^[^ ^6^ ^]^ Currently, electric‐driven air conditioning is the most commonly‐used method for controlling indoor temperature.^[^ ^7^ ^]^ However, the widespread use of air conditioners poses several challenges. For one, the proliferation of air conditioners leads to a significant increase in energy consumption and associated economic costs.^[^ ^8^ ^]^ Moreover, frequently‐used refrigerants, such as ozone‐depleting substances, contribute to ozone depletion, further exacerbating greenhouse gas emissions.^[^ ^9^ ^]^ Additionally, ≈2 to 4 billion people in low and middle‐income countries in tropical or subtropical regions do not have access to space‐cooling equipment.^[^ ^10^ ^]^ Therefore, it is urgent to seek alternative cooling strategies.

Passive Daytime Radiative Cooling (PDRC) technology has garnered extensive attention from scientists since the 20th century theoretically as it can effectively cool surfaces below ambient temperature without any additional energy consumption. This is achieved by reflecting most sunlight (within wavelength of 0.3–2.5 µm) and emitting long‐wave infrared (LWIR) radiation strongly to the cold outer space through the atmospheric transparency window (especially within wavelength of 8–13 µm).^[^ ^11^ ^]^ A large number of solid theoretical foundations and systematic studies have created great conditions for the birth of new materials for PDRC.^[^ ^11^, ^12^ ^]^ The countless innovative and practical‐applied PDRC materials have emerged over the last ten years, including but not limited to photonic crystal films,^[^ ^13^ ^]^ nanotextiles,^[^ ^14^ ^]^ cellulose nanocrystals,^[^ ^15^ ^]^ cooling woods,^[^ ^16^ ^]^ aerogel,^[^ ^17^ ^]^ pigment‐based paints^[^ ^18^ ^]^ and porous polymeric coatings.^[^ ^19^ ^]^ Up to now, the optical performance of PDRC materials under ideal conditions is almost reaching the theoretical limit.^[^ ^18^, ^19^, ^20^ ^]^ The key to PDRC relies on high solar reflectance (*R̅*_solar_ ≥ 0.9), as insufficient reflectance under strong sunlight causes severe heat gain, far greater than the heat loss through LWIR radiation.^[^ ^20^, ^21^ ^]^ Even if the material has perfect LWIR emittance (${\bar{\varepsilon }}_{\mathrm{LWIR}}$), a few percent solar absorption can rapidly heat the surface. However, this indispensable high solar reflectance is very likely to decline due to the environmental aging, making this technology ineffective after being exposed to outdoors for only a few months.^[^ ^18^, ^22^ ^]^ This limitation presents a significant challenge for the continuous development and application of PDRC technology.

To address this issue, researchers have begun to focus on the environmental durability of PDRC, considering aspects such as contamination‐resistance,^[^ ^14^, ^17^, ^18^, ^22^ ^]^ photothermal durability,^[^ ^18^, ^23^ ^]^ flame retardancy,^[^ ^15^, ^24^ ^]^ and acid and alkali resistance.^[^ ^25^ ^]^ We believe that this list will continue to grow, because the real‐world aging is more complex, contamination and weathering are usually synergistic. However, the commercialization of PDRC remains in its nascent stages due to its relatively late entry into the research arena, most reviews only provide basic theories, general materials and applications of PDRC,^[^ ^11^, ^12^, ^26^ ^]^ and there is currently no review focusing on environmental durability of PDRC. To bridge this gap, we show a comprehensive and detailed review of anti‐environmental aging PDRC (AEA‐PDRC) for the first time, aiming to establish a strong design and development work of AEA‐PDRC, and to break through the bottleneck restricting practical application in daily scenarios.

**Figure 1:**
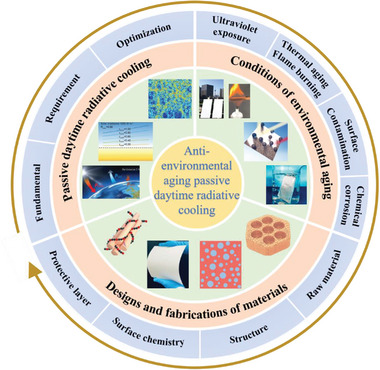
Overview of the mechanisms, environmental durability, designs and fabrications to construct anti‐environmental aging passive daytime radiative cooling. Reproduced with permission.^[^ ^11a^ ^]^ Copyright 2021, AAAS. Reproduced with permission.^[^ ^22b^ ^]^ Copyright 2022, Wiley‐VCH. Reproduced with permission.^[^ ^14a^ ^]^ Copyright 2021, American Chemical Society. Reproduced with permission.^[^ ^18c^ ^]^ Copyright 2022, Springer Nature. Reproduced with permission.^[^ ^19c^ ^]^ Copyright 2021, Springer Nature. Reproduced with permission.^[^ ^23^ ^]^ Copyright 2022, Wiley‐VCH.

**Figure** **1** provides an overview of the fundamentals, design strategies, material structures, and performance evaluations required for constructing AEA‐PDRC. This review is divided into four chapters to comprehensively address the subject matter. Chapter 1 discusses the optical mechanisms and optimizations of typical PDRC. Chapter 2 illustrates the intrinsic causes of PDRC failure due to environmental aging and corresponding fundamentals and characterization methods from four aspects, including UV exposure, thermal aging, surface contamination and chemical corrosion, providing guidance for the material selection and structural designs of AEA‐PDRC. Chapter 3 summarizes the works and developments of AEA‐PDRC materials, including design strategies, fabrications, structures, and performance evaluations. Notably, a single strategy can only achieve limited durability of one aspect of PDRC. Therefore, the general strategy of designing comprehensive AEA‐PDRC by combining several excellent durability properties is summarized and prospected in chapter 4 to promote the commercialization of PDRC techniques and enable PDRC materials long‐term working in harsh environments.

## 2 Passive Daytime Radiative Cooling

**Figure 2:**
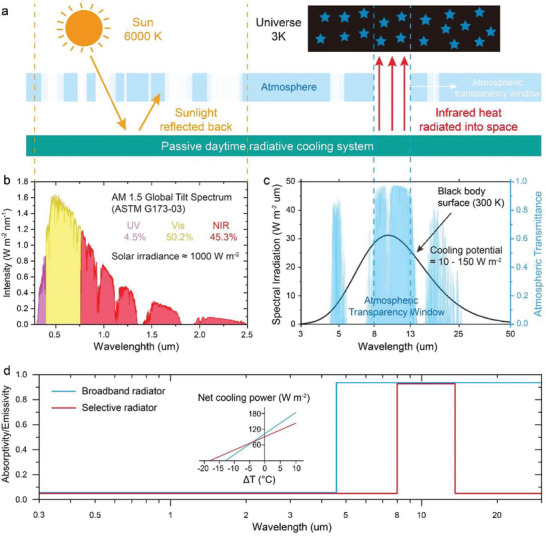
Passive daytime radiative cooling. a) Schematic diagram of the fundamental and working principle of PDRC. b) AM 1.5 standard solar spectrum based on ASTM G173‐03. c) Spectrum of a blackbody surface with a temperature of 300 K (solid black curve) and the atmospheric transparency window in the infrared regions (highlighted in blue background). d) Ideal emittance spectrum of broadband radiator and selective narrowband radiator. Inset showing the net radiative cooling power of two emitters as a function of the temperature difference between the emitter surfaces and their surroundings. c) Reproduced with permission.^[^ ^11c^ ^]^ Copyright 2019, AIP Publishing. d) Adapted with permission.^[^ ^11b^ ^]^ Copyright 2020, AAAS.

The achievement of PDRC is the first step before endowing anti‐environmental aging properties with PDRC. In systems of PDRC, thermal exchange with both the hot sun (≈5727 °C) and the cold outer space (≈−270 °C) are involved (**Figure** **2**a). The optical performance of used materials is crucial to achieve PDRC. In order to develop PDRC, it is essential to fully comprehend the importance of high solar reflectance (*R̅*_solar_ ≥ 0.9) and how to create structures with high *R̅*_solar_ and ${\bar{\varepsilon }}_{\mathrm{LWIR}}$. Therefore, we begin by discussing the fundamental physics of PDRC, including a detailed calculation of the cooling power's dependence on *R̅*_solar_ and ambient temperature to highlight the necessity of high *R̅*_solar_. The material selection and general optical design principles of PDRC are also summarized.

### 2.1 Fundamentals of Passive Daytime Radiative Cooling

All objects emit thermal radiation above absolute zero temperature, with higher temperatures resulting in greater amounts of radiation.^[^ ^27^ ^]^ The wavelength of the electromagnetic wave emitted is inversely proportional to the temperature of the objects. For instance, the sun's surface temperature is ≈5700 °C, with its peak radiation is in visible wavelengths (Figure 2b).^[^ ^28^ ^]^ Meanwhile, the temperature of most creatures on earth is usually between 20 and 40 °C, with the strongest radiation band in the mid/longwave infrared range (Figure 2c).^[^ ^11a^ ^]^ On the one hand, the atmosphere is transparent to electromagnetic waves within the range of 8–13 µm (atmospheric transparency window), which coincides with the thermal radiation band of most objects on earth (Figure 2c).^[^ ^11^, ^13^ ^]^ Therefore, the heat on earth can be directly transferred to outer space without additional energy cost through thermal radiation to achieve sub‐ambient cooling. On the other hand, different from nighttime radiative cooling, PDRC imposes stringent and demanding requirements on the *R̅*_solar_. This is because the solar energy power, which can reach up to ≈1000 W m^−2^, far exceeds the potential cooling power (≈10–150 W m^−2^) through atmospheric transparency windows,^[^ ^20b^ ^]^ making it significant to ensure that the peak solar absorbance does not exceed the energy amount emitted from the cooler. Specific quantitative requirements for *R̅*_solar_ of PDRC under different conditions will be outlined in Chapter 2.1.2. Therefore, high *R̅*_solar_ and high thermal infrared emittances are two necessary conditions for PDRC.

#### 2.1.1 Types of Thermal Emittances in Passive Daytime Radiative Cooling

When it comes to thermal infrared emittances, it is important to note that the object will absorb atmospheric counter radiation and thermal radiation from its surroundings outside the main atmospheric transparency window within wavelength of 8–13 µm, which can result in extra thermal gain that reduces cooling capacity.^[^ ^11c^ ^]^ However, atmospheric transparent channels distributed in other infrared bands can also provide additional cooling channels. For instance, cooling power in the long‐wave infrared band (20–25 µm) can effectively offset the parasitic heat gain, due to the high atmospheric transparency and weak atmospheric counter radiation within the band.^[^ ^18a^ ^]^ As such, broadband emitter (4–25 µm) and selective narrowband emitter (8–13 µm) have become the two ideal modes of PDRC (Figure 2d). The mode choice for a specific application depends on various factors such as the ambient temperature, surrounding environment, atmospheric conditions, and geographical locations, etc.^[^ ^29^ ^]^ Yin et al. gave the approximately linear response of the net radiative cooling power of the emitter in two modes as a function of the temperature difference between the emitter surfaces and their surroundings.^[^ ^11b^ ^]^ It can be seen that when the surface temperature is lower than the ambient temperature, the selective narrowband emitter can provide stronger passive cooling (Figure 2d). The broadband emitter has greater cooling power when the surface temperature is higher or close to the ambient temperature.

#### 2.1.2 Optical Requirements for *R̅*_solar_ of Passive Daytime Radiative Cooling

In order to quantitatively evaluate the requirements for *R̅*_solar_ of PDRC under different solar irradiance (*I*_solar_), there are two parameters to feel the cooling ability of PDRC intuitively, the sub‐ambient temperature drop and the cooling power. However, due to differences in test conditions, such as location, wind speed, and humidity, etc. sub‐ambient temperature drop rarely reflect reproducible results even for the same material from different tests.^[^ ^11c^ ^]^ In contrast, cooling power emerges as a relatively dependable and steadfast metric. Its reliability stems from its inherent capacity to accommodate environmental variables and its focus on the energy flow, minimizing disruptions caused by sudden temperature fluctuations.^[^ ^21b^ ^]^ Consequently, it provides a more consistent measure of PDRC's cooling performance.

Specifically, to estimate the cooling power of a radiative cooler, we can begin by analyzing the steady‐state heat transfer balance.^[^ ^11f^ ^]^ In general, for a radiative cooler at temperature *T* and ambient temperature *T*_a_ and exposed to the sky, the net cooling power *P*_net_ (*T*, *T*_a_) can be calculated as:

(1) $$\begin{array}{cc} P_{\mathrm{net}}\left(T,T_{a}\right) & =P_{\mathrm{rad}}\left(T\right)-P_{\mathrm{atm}}\left(T_{a}\right)-\left(1-{\bar{R}}_{\mathrm{solar}}\right)P_{\mathrm{solar}}\\ & \;-P_{\mathrm{nrad}}\left(T,T_{a}\right) \end{array}$$

where *P*_rad_ (*T*) is thermal radiation power from the cooler, *P*_atm_ (*T*_a_) is absorbed atmospheric longwave radiation power from atmospheric thermal radiation, *P*_solar_ is absorbed solar irradiance and *P*_nrad_ (*T*, *T*_a_) is the nonradiative heat transfer, including conduction and convection, between the cooler and the surrounding environment. In practice, a heater can be used to compensate the heat loss of the cooling device to make its temperature equal to the ambient one. Then, the *P*_net_ (*T*, *T*_a_) turns *P*_net_ (*T*_a_, *T*_a_) and can be further defined as *P*_cool_ (*T*_a_), which equals to the heat power. The *P*_rad_ (*T*) becomes *P*_rad_ (*T*_a_) and the *P*_atm_ (*T*) becomes *P*_atm_ (*T*_a_) and can be described as

(2) $$P_{\mathrm{rad}}\left(T_{a}\right)=2\pi =\int_{0}^{\frac{\pi }{2}}\cos\theta \sin\theta \int_{0}^{\infty }I_{\mathrm{bb}}\left(T_{a},\lambda \right)\varepsilon \left(\lambda ,\theta \right)d\lambda d\theta$$

(3) $$\begin{array}{cc} P_{\mathrm{atm}}\left(T_{a}\right) & =2\pi \int_{0}^{\frac{\pi }{2}}\cos\theta \sin\theta \\ & \;\times \int_{0}^{\infty }I_{\mathrm{bb}}\left(T_{a},\lambda \right)\varepsilon \left(\lambda ,\theta \right)\varepsilon_{a}\left(\lambda ,\theta \right)d\lambda d\theta \end{array}$$

(4) $$\varepsilon_{a}\left(\lambda ,\theta \right)=1-t_{a}{\left(\lambda ,0\right)}^{1/\cos\theta }$$

where ε(λ, θ) and ε_a_(λ,θ) are spectral and angular emittance of the radiative cooling surface and ambient air, *t*_a_ (*λ*, 0) is the atmospheric transmittance at the zero zenith angle. The data of *t*_a_ (*λ*, 0) is from ATRAN modeling software in our estimation.

For a radiative cooler with a unit area of 1 m^2^, *P*_nrad_ (*T*, *T*_a_) can be expressed as *h*_c_ (*T*_a_ – *T*) by introducing a non‐radiative heat transfer coefficient *h*_c_ (unit of W m^−2^ K^−1^). It is worth noting that the value of *h*_c_ can vary significantly, ranging from 2 to 20 W m^−2^ K^−1^,^[^ ^11c^ ^]^ depending on the specific environmental conditions. To mitigate the impact of this uncontrolled factor, it is advantageous to set Δ*T*  =  *T*_a_ – *T  =* 0, thus eliminating the *P*_nrad_ (*T*, *T*_a_) term from consideration.

The ideal blackbody radiation without solar absorption can be created by setting 100% *R̅*_solar_ and 100% ${\bar{\varepsilon }}_{\mathrm{LWIR}}$. Then *P*_cool_ (*T*_a_) can be written as

(5) $$\begin{array}{cc} P_{\mathrm{cool}}\left(T_{a}\right) & =2\pi \int_{0}^{\frac{\pi }{2}}\cos\theta \sin\theta \\ & \;\times \int_{8\;m}^{13\mu m}I_{\mathrm{bb}}\left(T_{a},\lambda \right)\varepsilon \left(\lambda ,\theta \right)t_{a}{\left(\lambda ,0\right)}^{1/\cos\theta }d\lambda d\theta \end{array}$$

It is noted that *P*_cool_ as a function of ambient temperature *T*_a_ under different *R̅*_solar_ can be simply gained by resetting non‐zero term of (1 − *R̅*_solar_) *P*_sun_ to obtain the net cooling energy *P*_net_ (*T*_a_):

(6) $$P_{\mathrm{net}}\left(T_{a}\right)=P_{\mathrm{cool}}\left(T_{a}\right)-\left(1-{\bar{R}}_{\mathrm{solar}}\right)P_{\mathrm{sun}}$$

**Figure 3:**
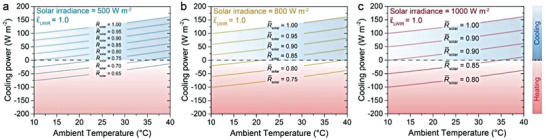
Net cooling power as a function of ambient temperature for various *R̅*_solar_ under different solar irradiance: a) 500 W m^−2^, b) 800 W m^−2^ and c) 1000 W m^−2^. Passive cooling is difficult to achieve when *R̅*_solar_ < 0.9 under the strong sunlight even if the material has a perfect LWIR emittance.

**Figure** **3** depicts the cooling power curve as a function of *R̅*_solar_ and ambient temperature under the varying solar irradiances. Cooling power greater than 0 indicates that the radiative cooler is still effective in cooling, whereas cooling power less than 0 suggests the occurrence of undesirable heating effect.^[^ ^11f^ ^]^ It is evident that the positive cooling power demands higher *R̅*_solar_ as the solar irradiance energy increases. Specifically, *R̅*_solar_ of the radiative cooler is typically greater than 0.90 for sub‐ambient cooling when the solar power exceeds 1000 W m^−2^.

In summary, from the perspective of optical design, PDRC technology is generally divided into two combinations: “high *R̅*_solar_ + high selective narrowband infrared emittance (8–13 µm)” and “high *R̅*_solar_ + high broadband infrared emittance (4–25 µm)”. The aforementioned analysis highlights two significant issues. First, designing PDRC materials with high *R̅*_solar_ is of utmost importance to ensure optical performance under ideal conditions. Second, the AEA‐PDRC materials must prioritize maintaining high *R̅*_solar_ to guarantee the long‐term effectiveness in harsh environments.

### 2.2 Optical Optimization of Passive Daytime Radiative Cooling

In this section, we categorize PDRC materials based on optics, specifically their ability to achieve high *R̅*_solar_ and ${\bar{\varepsilon }}_{\mathrm{LWIR}}$. Our focus is on two major categories of paint‐based models: scatterers‐embedded matrix and porous structure. Both of them provide great potential for large‐scale manufacturing, as they are easy to construct and have become ideal for further endowing anti‐environmental aging properties.^[^ ^18^, ^20^, ^22^, ^30^ ^]^ In particular, the model of porous structure model has shown promising results and has increased the optical performance to state‐of‐the‐art levels.^[^ ^19^, ^26^, ^31^ ^]^ Due to the diversity of design concepts and manufacturing techniques, the above classification is sometimes incomplete and inaccurate, and is for reference only.

#### 2.2.1 Scatterers‐Embedded Matrix

**Figure 4:**
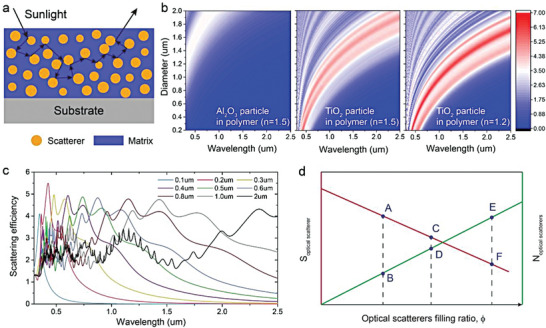
Scatterers‐embedded matrix. a) Schematic diagram of scatterers embedded in a matrix. b) The scattering efficiency of a single spherical particle as a function of particle diameter over wavelength range of 0.3–2.5 µm in different scattering media. c) The calculated scattering efficiency as a function of scatterer size and incident light wavelength. The strong scattering peaks from scatterers with various sizes cover the whole solar spectrum to support the high *R̅*_solar_. d) Scattering strength of one optical scatterer (red line) and scatterer concentration (green line) as a function of the filling ratio (*ϕ*) of scatterers.

In the 20th century, high albedo white roof coatings based on pigments embedded in resin became commercially available in the industry,^[^ ^11e^ ^]^ laying the foundation for the conceptual design of PDRC. While these materials cannot directly achieve sub‐ambient cooling under strong sunlight due to their moderate *R̅*_solar_, the fundamental structure, hybrid optical scatterers embedded in matrix, represent an ideal structural design of PDRC. In order to achieve high *R̅*_solar_ based on this structure, a thorough understanding of the interaction between light and optical scatterers is required. According to Snell's law, the large refractive index difference between two different media triggers strong light scattering, leading to high reflectance (**Figure** **4**a). To maximize *R̅*_solar_, three significant issues must be considered: the selection of materials with different refractive indexes, the size distribution and the filling ratio of optical scatterers.

In order to further explain quantitatively, Mie theory calculated from Maxwell equations describes the cross‐sectional area of a homogenous spherical scatterer, the size of the scatterer, the refractive index of the scatterer and the matrix, which provides excellent guidance for understanding the relationship between the scattering efficiency of a single scatterer and the reflectance of derived structure. The scattering cross‐section (*C*_sca_) of a spherical scatterer is determined as:^[^ ^32^ ^]^

(7) $$C_{\mathrm{sca}}=\frac{2\pi }{k^{2}}\sum_{n=1}^{\infty }\left(2n+1\right)\left({\left|a_{n}\right|}^{2}+{\left|b_{n}\right|}^{2}\right)$$

(8) $$a_{n}=\frac{m\psi_{n}\left(mx\right)\psi_{n}^{\prime }\left(x\right)-\psi_{n}\left(x\right)\psi_{n}^{\prime }\left(mx\right)}{m\psi_{n}\left(mx\right)\xi_{n}^{\prime }\left(x\right)-\xi_{n}\left(x\right)\psi_{n}^{\prime }\left(mx\right)}$$

(9) $$b_{n}=\frac{\psi_{n}\left(mx\right)\psi_{n}^{\prime }\left(x\right)-m\psi_{n}\left(x\right)\psi_{n}^{\prime }\left(mx\right)}{\psi_{n}\left(mx\right)\xi_{n}^{\prime }\left(x\right)-m\xi_{n}\left(x\right)\psi_{n}^{\prime }\left(mx\right)}$$

here *k* is wavevector, *n* is multipole order, *a_n_* is *n*th electric mode coefficient, *b_n_* is *n*th magnetic mode coefficient, *m* is the relative refractive index, *ψ* and *ξ* are Riccati‐Bessel functions. Then the scattering efficiency coefficient (*Q*_sca_) is the normalization of *C*_sca_ as:

(10) $$Q_{\mathrm{sca}}=\frac{C_{\mathrm{sca}}}{\pi r^{2}}$$

here *πr* ^2^ is the geometrical cross‐sectional area of the scatterer.

To illustrate the principles of material selection for designing PDRC, the scattering efficiency of a single scatterer with different refractive indexes in various media were calculated. As shown in Figure 4b, the results demonstrated that scatterers with higher refractive index differences relative to the polymer matrix (*n* = 1.5), such as titanium dioxide (TiO_2_) (*n* ≈ 2.5) spheres, can scatter sunlight more strongly compared with aluminum oxide (Al_2_O_3_) (*n* ≈ 1.76) spheres in the same polymer matrix (*n* = 1.5). Additionally, scatterers in a medium with a lower refractive index can also accomplish higher scattering efficiency. However, the loss of *R̅*_solar_ caused by intrinsic absorption must be considered when selecting a scatterer, as relatively narrow‐bandgap materials like TiO_2_ can absorb ultraviolet light.^[^ ^20b^ ^]^ Therefore, materials with wider bandgaps, such as Al_2_O_3_ (7.0 eV), barium sulfate (BaSO_4_) (6.0 eV), calcium carbonate (CaCO_3_) (7.0 eV) etc., are also viable candidates for optical scattering materials.

Second, the size of the scatterer, depending on the refractive index difference with the medium and the wavelength of the scattered light, is also critical for the reflectance of the entire system.^[^ ^14^, ^18^ ^]^ For instance, TiO_2_ spheres with size distributions between 100 and 2000 nm embedded in matrix (*n* = 1.5) can utilize multiple Mie resonances to generate the scattering peak required to efficiently cover the entire visible‐to‐near‐infrared band (Figure 4c).

Lastly, the effect of the filling ratio of scatterers in the medium on the optical performance of the system is a complex issue. Different filling ratios will inevitably change the spacing between scatterers and affect the total scattering efficiency. Briefly, low filling ratios result in independent scattering, which guarantees the scattering efficiency of a single scatterer but decreases the number of scattering interfaces between scatters and medium. In contrast, high filling ratios increase the scattering interfaces and times, but the crowding can lead to dependent scattering, thereby reducing the scattering efficiency of a single scatterer.^[^ ^33^ ^]^ Therefore, an optimal filling ratio should be determined to balance these factors. In Figure 4d, we describe the competition between the scattering efficiency of a single scatterer (*S*_optical scatterer_) and the number of scatterers (*N*_optical scatterer_) as the filling ratio (*ϕ*) increases. The definition of scatterer filling ratio (*ϕ*) is as follows:

(11) $$\;\phi_{\mathrm{sca}}=\frac{V_{\mathrm{sca}}}{V_{\mathrm{sca}}+V_{\mathrm{polymeric}\;\mathrm{binder}}+V_{\mathrm{other}\;\mathrm{fillers}}}$$

Here, *V*_sca_, *V*_polymeric binder_, *V*_other fillers_ are volume of scatters, polymeric binder and all other fillers respectively. The total scattering efficiency of the coating (*S*_coating_) is proportional to *S*_optical scatterer_ and *N*_optical scatterer_. We can qualitatively define:

(12) $$S_{\mathrm{coating}}∼S_{\mathrm{optical}\;\mathrm{scatterer}}\times \;N_{\mathrm{optical}\;\mathrm{scatterer}}$$

when *ϕ* is low, S≈*A* × *B*, although *A* is high, *B* is low, and S may not be desirable. Likewise, dependent light scattering effect caused by high *ϕ* will inevitably result in low *S*_optical scatterer_, leading to a relatively low *S*_coating_ (*S*_coating_≈*E* × *F*). Therefore, the ideal high *S*_coating_ must be achieved at an appropriate medium filling ratio (such as point C and D in Figure 4d).

While empirical formulas can provide qualitative guidance for designing PDRC, precise quantitative experimental parameters rely on modeling and calculation through optical simulation software tailored to specific materials and structures. For example, Li et al. prepared a BaSO_4_ particles‐embedded acrylic coating and improved its *R̅*_solar_ to 0.981 using a modified Lorentz‐Mie theory‐based Monte Carlo simulation.^[^ ^18b^ ^]^ Similarly, the Monte Carlo simulation also assisted Zeng et al. in optimizing the structural parameters of a TiO_2_‐polylactic acid multilayer PDRC metafabric.^[^ ^14a^ ^]^ In addition, the Finite‐Difference Time‐Domain (FDTD) algorithm is also widely‐used to design optical structures and determine parameters in PDRC.^[^ ^18^, ^19^ ^]^ These simulation tools enable researchers to gain insights into the optical properties of PDRC and help in the optimization of radiative cooler.

#### 2.2.2 Porous Structure

From an optical perspective, the mesoporous structure is similar to the structure of scatterers‐embedded matrix. Because light scattering is a physical phenomenon that is independent of chemistry, air can serve as an ideal scatterer to replace pigments due to its ultra‐low refractive index and almost zero ultraviolet light absorption, maximizing light scattering efficiency.^[^ ^20b^ ^]^ The major structural parameters are pore size, porosity, and pore distribution.

**Figure 5:**
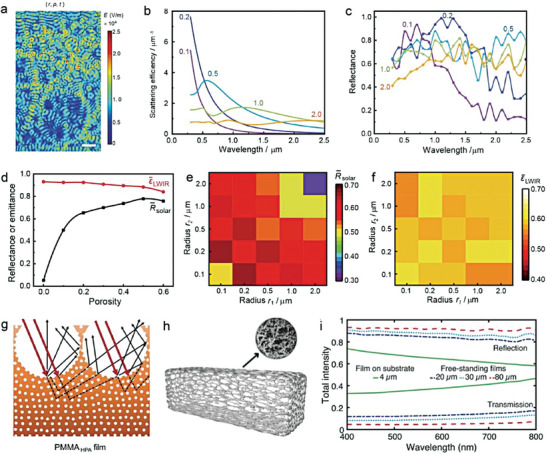
Porous structure. a) The geometry of a porous structure in simulation (scale bar = 2 µm). b) Simulated scattering efficiency and c) simulated reflectance spectra of pores with different diameters in P(VdF‐HFP) matrix. d) Calculated *R̅*_solar_ and ${\bar{\varepsilon }}_{\mathrm{LWIR}}$ with different porosity. e,f) Maximum e) *R̅*_solar_ and f) ${\bar{\varepsilon }}_{\mathrm{LWIR}}$ for the porous P(VdF‐HFP) model at different combinations of dual‐scale pores with two different radii. g) Schematic of micro‐nano dual‐scale pores. h) Structure diagram of porous PMMA coating. i) Transmission and reflection spectrums of porous PMMA films with different thickness. a–f) Reproduced with permission.^[^ ^34^ ^]^ Copyright 2021, American Chemical Society. g) Reproduced with permission.^[^ ^19c^ ^]^ Copyright 2021, Springer Nature. h,i) Reproduced with permission.^[^ ^15c^ ^]^ Copyright 2018, Wiley‐VCH.

To gain a better understanding of these parameters, A numerical simulation model based on porous P(VdF‐HFP) was developed by Chen et al.^[^ ^14a^ ^]^ The authors employed a 2D porous structure for modeling to reduce the calculation load (**Figure** **5**a). This was mainly due to the dependence of total reflectance on wavelength, which can be explained by the pore size and porosity. When the pore size is small in a porous coating (e.g., 0.1 µm radius), the pores are relatively more numerous and denser. Shorter wavelengths of sunlight, including the high‐energy ultraviolet and visible light, possess smaller or comparable wavelengths than the pore size, allowing them to easily enter the air‐voids within the coating and interact with the air‐solid interfaces.^[^ ^33^ ^]^ Within these smaller pores, a captivating phenomenon unfolds as shorter wavelengths of light undergo multiple rounds of scattering and reflection, culminating in elevated reflectance. In contrast, sunlight carrying longer wavelengths, typified by the near‐infrared light, bears diminished energy levels. These extended wavelengths gracefully transcend the pore dimensions, facilitating their unhindered traversal through the smaller pores, with minimal scattering or absorption.^[^ ^32^ ^]^ The outcome is a reduction in reflectance for light featuring longer wavelengths within coatings with smaller pore sizes, courtesy of their unimpeded journey through the coating. A similar dynamic is at play when the pore size within the porous coating are expanded. Sunlight bearing wavelengths corresponding to the enlarging pores becomes increasingly prone to multiple scattering and reflection events, driving the reflection peak toward longer wavelengths in harmony with aperture growth. Conversely, shorter‐wavelength sunlight encounters fewer impediments, enabling it to traverse the coating with ease and leading to reduced reflectance within the shorter wavelength range. The classical Mie scattering theory also demonstrated that the scattering peak significantly redshifts with the increase of pore radius, and the maximum scattering efficiency occurs at a wavelength slightly larger than the pore radius. The pore radii between 0.1–0.5 µm exhibited the strongest scattering efficiency for the wavelength with the highest solar energy (0.4−0.7 µm), with 0.2 µm being the optimal radius to improve *R̅*_solar_ (Figure 5b,c). The ${\bar{\varepsilon }}_{\mathrm{LWIR}}$ almost stayed unchanged with the change of radius, mainly due to the high emissivity/absorptance of P(VdF‐HFP) in the atmospheric transparency window. Second, porosity is another core parameter that affects optical performance, and more pores are able to scatter sunlight more effectively (Figure 5d). However, too many pores may increase light transmission and slightly reduce *R̅*_solar_. On the other hand, more pores mean a decrease in polymer content, weakening the absorptance in the longwave infrared region, resulting in a slight fall of ${\bar{\varepsilon }}_{\mathrm{LWIR}}$. Lastly, the dual‐scale uneven distribution of micro and nano pores can trigger the collective behavior of multiple Mie behaviors to cover the entire solar spectrum, further strengthening the *R̅*_solar_ (Figure 5e,f).

These results well illustrated general principles of parameter adjustment in porous structural design. Qualitatively, these laws are also applicable to other various media. Wang et al. demonstrated that the combination of ordered symmetric micropores (≈4.6 µm diameter) and randomized nanopores (≈250 nm average diameter) in polymethyl methacrylate (PMMA) could achieve up to a *R̅*_solar_ of 0.95 (Figure 5g).^[^ ^14a^ ^]^ Among them, ≈4.6 µm micropores effectively scattered sunlight at ultraviolet‐visible‐near infrared wavelength, while the rich and random ≈250 nm nanopores greatly reduced the average scattering path and transmission through the bulk, which further enhanced scattering at shorter visible wavelength. Furthermore, inspired by the anisotropic chitin network in the scales covering the Cyphochilus beetles, Syurik et al. optimized the high scattering porous white network composed of PMMA (Figure 5h), and the visible light reflectance of a only 4 µm thick film could reach 0.75 (Figure 5i).^[^ ^15c^ ^]^ With the increase of thickness, the reflectance/transmittance of the porous PMMA film also increased/decreased significantly, which was mainly attribute to more reflections and refractions at the air‐solid interfaces.

In conclusion, this chapter is the basis for the design of PDRC. On the foundation of ensuring high *R̅*_solar_ and ${\bar{\varepsilon }}_{\mathrm{LWIR}}$, environmental durability can be further added to PDRC.

## 3 Conditions of Environmental Aging

Despite over nearly decades of intense researches, PDRC is, however, still plagued with environmental aging problems that severely limit their real‐world applications, caused by UV radiation, high and low temperature exposure, surface contamination and acid/alkali erosion.^[^ ^11^, ^12^, ^22^, ^29^ ^]^ First of all, we review the intrinsic causes of failure of PDRC materials due to UV exposure, thermal aging, and chemical corrosion, which are fundamental and necessary for further designing high‐performance AEA‐PDRC materials. In addition, soiling‐induced degradation of *R̅*_solar_ is a primary challenge for PDRC. To improve the contamination resistance and maintain high *R̅*_solar_, different super‐wetting surfaces, including liquid‐repellent surfaces with moderate hydrophobicity,^[^ ^14^, ^19^ ^]^ superhydrophobicity^[^ ^16^, ^17^, ^18^, ^22^ ^]^ and superamphiphobicity,^[^ ^35^ ^]^ and photocatalysis‐induced hydrophilic surfaces^[^ ^36^ ^]^ have been proposed. Therefore, we provide detailed descriptions on liquid‐solid interfaces and wetting states, dust particle adhesion on surfaces and self‐cleaning mechanisms. Lastly, it is worth briefly discussing the characterization methods used in the literature for the aforementioned environmental aging conditions, as different characterizations may affect fair comparisons between different works. We also recommend some standard testing methods.

### 3.1 Ultraviolet Exposure

The ultraviolet (UV) aging of PDRC poses a significant challenge that demands the attention of researchers. First, unlike nighttime radiative cooling, PDRC must cool during the daytime, which exposes it to intense sunlight irradiance for prolonged periods.^[^ ^11^, ^26^ ^]^ As a result, UV exposure is an objective and inevitable external factor that brings a persistent threat to PDRC materials. Additionally, most polymer‐based materials tend to absorb UV energy under sunlight, leading to the dissociation of molecular chains and the initiation of free radical chain reactions.^[^ ^37^ ^]^ This process can result in a decline in the relative molecular mass, tensile strength, and modulus of the material.^[^ ^38^ ^]^ Moreover, the molecular chain fracture and free radical reaction resulting from UV aging will severely affect the ${\bar{\varepsilon }}_{\mathrm{LWIR}}$ depending heavily on the molecular‐level design of chemical bonding.^[^ ^19^, ^22^ ^]^ In general, materials with lower bond energy are more susceptible to be broken under UV energy.

**Figure 6:**
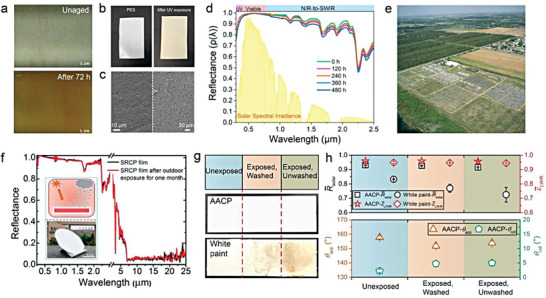
Ultraviolet exposure. a) Surface color changes of thermoplastic polyurethane with UV aging. b) Photographs of PES film before and after UV exposure. c) SEM images of PES film before (left) and after the real‐world exposure (right). d) Reflectance spectrum of porous PMMA films with the different accelerated weathering time. e) Florida's outdoor weathering site. f) The spectrum and appearance of superhydrophobic PDRC film after being placed outdoors for one month. g) Photos of superhydrophobic PDRC coating and commercial white paint coating after 6 months of real‐world exposure. f) Comparison of optical and wetting properties before and after real‐world exposure. a) Reproduced with permission.^[^ ^39a^ ^]^ Copyright 2010, Elsevier. b,c) Reproduced with permission.^[^ ^42^ ^]^ Copyright 2023, Wiley‐VCH. d,e) Reproduced with permission.^[^ ^19c^ ^]^ Copyright 2021, Springer Nature. f) Reproduced with permission.^[^ ^22c^ ^]^ Copyright 2021, Royal Society of Chemistry. g,h) Reproduced with permission.^[^ ^18c^ ^]^ Copyright 2022, Springer Nature.

Second, the yellowing effect caused by UV degradation of polymers is unacceptable for PDRC, as it irreversibly damages the ultra‐white appearance and reduces *R̅*_solar_, rendering PDRC ineffective. Long‐term UV exposure of polymers leads to photooxygen aging, where weak molecular bonds on the polymer chain break under UV irradiation, forming free radicals that react with oxygen to produce aldehydes, peroxides, and other substances, resulting in yellowing.^[^ ^39^ ^]^ This is a common phenomenon that has been observed by many researchers. Boubakri et al. reported that thermoplastic polyurethane changed from colorless to yellow within a few hours of UV aging, gradually turning brown with time (**Figure** **6**a).^[^ ^39a^ ^]^ This color shifting can be attributed to oxidation reactions which lead to the formation of an oxidized layer on the surface. Zhao et al. found that ethylene propylene glycol monomer (EPDM) turned red, yellow, and light during UV aging, with specular gloss initially increasing and then decreasing with time.^[^ ^40^ ^]^ Woo et al. also demonstrated the yellowing effect of epoxy–organoclay nanocomposites under UV exposure.^[^ ^41^ ^]^ Li et al. demonstrated that the polyethersulfone (PES) film for PDRC turned yellow after 1000 h of UV exposure (Figure 6b), and SEM images showed that the morphology of the PES film was fragmented after 7 days of strong sunlight exposure (Figure 6c).^[^ ^42^ ^]^ Similarly, Wang et al. conducted accelerated weathering test on porous PMMA film for 480 h and noted that the weak chemical bond energy of C─C and C─H in common polymer binders made them vulnerable to slight impacts on *R̅*_solar_ due to long‐term UV irradiation (Figure 6d).^[^ ^19c^ ^]^

Lastly, the two most common characterization methods for evaluating the UV resistance of PDRC materials are using UV lamp irradiation and direct sunlight exposure in the real world. On the one hand, the UV lamp irradiation method can accurately control variables, eliminate interference from other factors, and evaluate the UV resistance of materials solely.^[^ ^14^, ^18^, ^22^, ^23^ ^]^ The irradiation of UV lamp usually requires consideration of testing parameters, including but not limited to the wavelength and intensity of UV radiation, exposure time, and the distance between the lamp and the sample surface.^[^ ^43^ ^]^ One of the key benefits of this laboratory‐level accelerated aging test method is its capacity to facilitate fair and quantitative comparisons of material properties across various studies. For instance, Florida is an internationally recognized benchmark location for outdoor exposure testing due to its high intensity of sunlight, sufficient annual ultraviolet radiation and high temperatures throughout the year (Figure 6e). Sleiman et al. mentioned that accelerated exposure (0.89 W m^−2^ at 340 nm) in the Q‐lab UV Tester (QUV) for 1000 h could be approximately equivalent to 1 year of Florida sunshine (about annual UV dosage = 275 MJ m^−2^),^[^ ^43^, ^44^ ^]^ providing a standard accelerated aging testing method that could be performed in the laboratory. On the other hand, exposure to sunlight in the real world is also an effective characterization method that more realistically reflects the photothermal durability of PDRC materials, and has been adopted in many studies.^[^ ^18^, ^19^, ^22^ ^]^ Liu et al. exposed the PDRC material to outdoor conditions for a duration of one month and then measured the *R̅*_solar_ (Figure 6f). Wang et al. conducted a 40 days exposure test on a PDRC film, which was installed on a rooftop in Shanghai to ensure that the film's surface was fully exposed to the natural environment, facing the sky. However, it should be noted that test results obtained through this method are often influenced by multiple aging factors, such as soiling and weathering, which can act synergistically in nature.^[^ ^11^, ^18^ ^]^ For instance, Song et al. conducted a comprehensive study on the real‐world aging performance of superhydrophobic PDRC coatings and commercial white paint coatings. All coating slides were divided into three regions: unexposed/(exposed, washed)/(exposed, unwashed). Each region was individually characterized after real‐world test (Figure 6g,h). The exposed samples were subjected to natural weathering conditions, including but not limited to sunlight irradiation, high temperatures, rainwater, and contamination, for a period of ≈6 months, spanning three seasons. Moreover, differences in sunlight intensity due to geographical locations can cause significant variations in test outcomes.^[^ ^29^ ^]^ Therefore, compared to the UV lamp irradiation method, this characterization method is difficult to directly demonstrate the single UV resistance of materials.

Overall, the laboratory UV accelerated aging testing is necessary to demonstrate the UV durability of PDRC materials, which is a more convincing characterization method. Usually, it should be based on certain standards, such as American Society for Testing and Materials (ASTM) standard, and can be equivalently converted to real‐world sunlight irradiation. Because the low light source intensity or insufficient exposure time, the real‐world sunlight exposure testing may not be able to evaluate the true performance of PDRC materials. Nevertheless, this testing remains an indispensable tool for comprehensive characterization of various aspects of PDRC materials after conducting accelerated aging tests on a single aspect.

### 3.2 Thermal Aging and Flame Burning

Thermal aging refers to the potential deterioration of PDRC materials when exposed to high temperatures or direct contact with flames. Similar to UV exposure, thermal aging involves oxidation reactions,^[^ ^45^ ^]^ thermal crosslinking^[^ ^46^ ^]^ and free radical reactions,^[^ ^47^ ^]^ leading to a decline in physical and chemical properties. Structural damage and changes in chemical composition can greatly impact the mechanical properties and lifespan of materials, while surface color changes can be detrimental to the high *R̅*_solar_ of PDRC. Fortunately, outdoor temperatures generally remain below 70 °C,^[^ ^48^ ^]^ and even after being heated by sunlight, the temperature of white surfaces typically does not exceed 80 °C.^[^ ^49^ ^]^ This is not a significant threat to most polymers suitable for PDRC. Thus far, there have been few reports of PDRC materials failing or experiencing substantial performance degradation due to high‐temperature environments. However, it is important to consider the accelerating effect of thermal aging when combined with other photoaging factors, such as UV exposure, as they often act synergistically.^[^ ^50^ ^]^ Additionally, special attention should be given to the direct flame burning of PDRC materials under certain circumstances.^[^ ^15^, ^24^, ^51^ ^]^ Flame temperatures can range from a few hundred to several thousand degrees, which is unacceptable for the majority of polymer‐based PDRC materials. Inorganic PDRC materials have demonstrated significant advantages toward flame retardancy, as highlighted in the researches conducted by Tsai et al^[^ ^51^ ^]^ and Chen et al^[^ ^15a^ ^]^ in 4.2.2.

In terms of performance characterization, the commonly‐employed method for PDRC materials is to place them in a thermal aging test chamber or on a heating platform for a specific duration and measure their optical performance after testing to check cooling capacity.^[^ ^18^, ^19^, ^25^, ^52^ ^]^ Maintaining proper humidity levels during testing is crucial because elevated humidity can expedite chemical reactions, such as hydrolysis, creep, and dissolution, thereby accelerating material aging and degradation.^[^ ^53^ ^]^ As previously mentioned, single high‐temperature environment restricted direct aging effects on PDRC materials. Instead, they are more likely to act as synergistic factors to catalyze other environmental aging factors. Consequently, long‐term monitoring of PDRC samples in real‐world remains an ideal approach to comprehensively evaluate environmental durability, particularly with regards to resistance against photothermal aging, which has more practical significance.

### 3.3 Surface Contamination

In reality, even small amounts of natural pollutants deposited on the surface of PDRC materials will inevitably absorb sunlight, significantly reducing *R̅*_solar_ and thus compromising cooling capacity. Thus, it is essential to develop effective methods to remove pollutants. Conventional dedusting techniques, such as ultrasonic washing and mechanical cleaning are active methods that require energy input and are labor‐intensive. These methods can lead to the wastage of water resources, the damage of materials, and higher economic costs. A promising alternative is the use of a passive technology, such as self‐cleaning based on liquid‐repellent surfaces.^[^ ^43^, ^54^ ^]^ Such surfaces enable droplets to pick up solid contaminants and roll off rapidly. To build liquid‐repellent surfaces and gain insights into the self‐cleaning process, we present the different solid‐liquid wetting states, the adhesion of soiling particles, and the dust removal from self‐cleaning liquid repellent surfaces. Additionally, the photocatalytic self‐cleaning technology that can convert organic pollutants into carbon dioxide and water is also introduced.

#### 3.3.1 Liquid‐Solid Interfaces and Wetting States

When the liquid wetting of a solid surface occurs in air, a triple‐phase interface consisting of solid, air and liquid is built, and a contact angle that is defined as the angle between the tangent to the liquid–air interface and the solid surface at the three‐phase contact line can be observed. The classical Young's equation describes the relationship between the static contact angle (θ_*Y*_) and the interfacial tension:^[^ ^55^ ^]^

(13) $$\cos\theta_{Y}=\frac{\gamma_{SA}-\gamma_{SL}}{\gamma_{LA}}$$

Here, γ_*SA*_, γ_*SL*_ and γ_*LA*_ are the interfacial tensions at the solid‐air, solid‐liquid and liquid‐air interfaces, respectively. It can predict the following wetting conditions: when θ_*Y*_ = 0, completely wetting; when θ_*Y*_ < 90°, partially wetting; when θ_*Y*_ > 90°, non‐wetting; when θ_*Y*_ = 180°, completely non‐wetting. θ_*Y*_ = 90° is the boundary between non‐wettability and wettability. By gradually increasing or decreasing the volume of a droplet, a maximal contact angle, namely the advancing angle, θ_*adv*_ and a minimal contact angle, namely the receding angle, θ_*rec*_ can be observed respectively. θ_*adv*_ −  θ_*rec*_ is defined as the contact angle hysteresis, which affects the sliding angle, θ_*slid*_or roll‐off angle, θ_*roll*_ represents how easily a droplet can slide or roll on a solid surface. The quantitative relationship between θ_slid_ or θ_roll_, θ_adv_ and θ_rec_ is as follows:^[^ ^56^ ^]^

(14) $$mg\left(\sin\left(\theta_{\mathrm{slid}}\;\mathrm{or}\;\;\theta_{\mathrm{roll}}\right)\right)/\omega =\gamma \left(\cos\theta_{\mathrm{rec}}-\cos\theta_{\mathrm{adv}}\right)$$

where *m* is the droplet mass, *g* is the acceleration of gravity, ω is the droplet diameter (the width of the contact surface between the droplet and the surface in the vertical sliding (rolling) direction), and γ is the surface tension.

To modify the Young's model by considering the surface roughness, the Wenzel model was introduced. The roughness factor (*r*) can be described as:^[^ ^57^ ^]^

(15) $$r=\frac{\mathrm{actual}\;\mathrm{surface}\;\mathrm{area}}{\mathrm{projected}\;\mathrm{surface}\;\mathrm{area}}$$

In the Wenzel model, it is assumed that there is no air at the interface between the solid surface and liquid, and the rough protrusions on the solid surface are uniformly distributed. Wenzel proposed an intuitive equation to describe the apparent contact angle (θ_*w*_) of the rough surface:

(16) $$\cos\theta_{w}=r\cos\theta_{Y}$$

**Figure 7:**
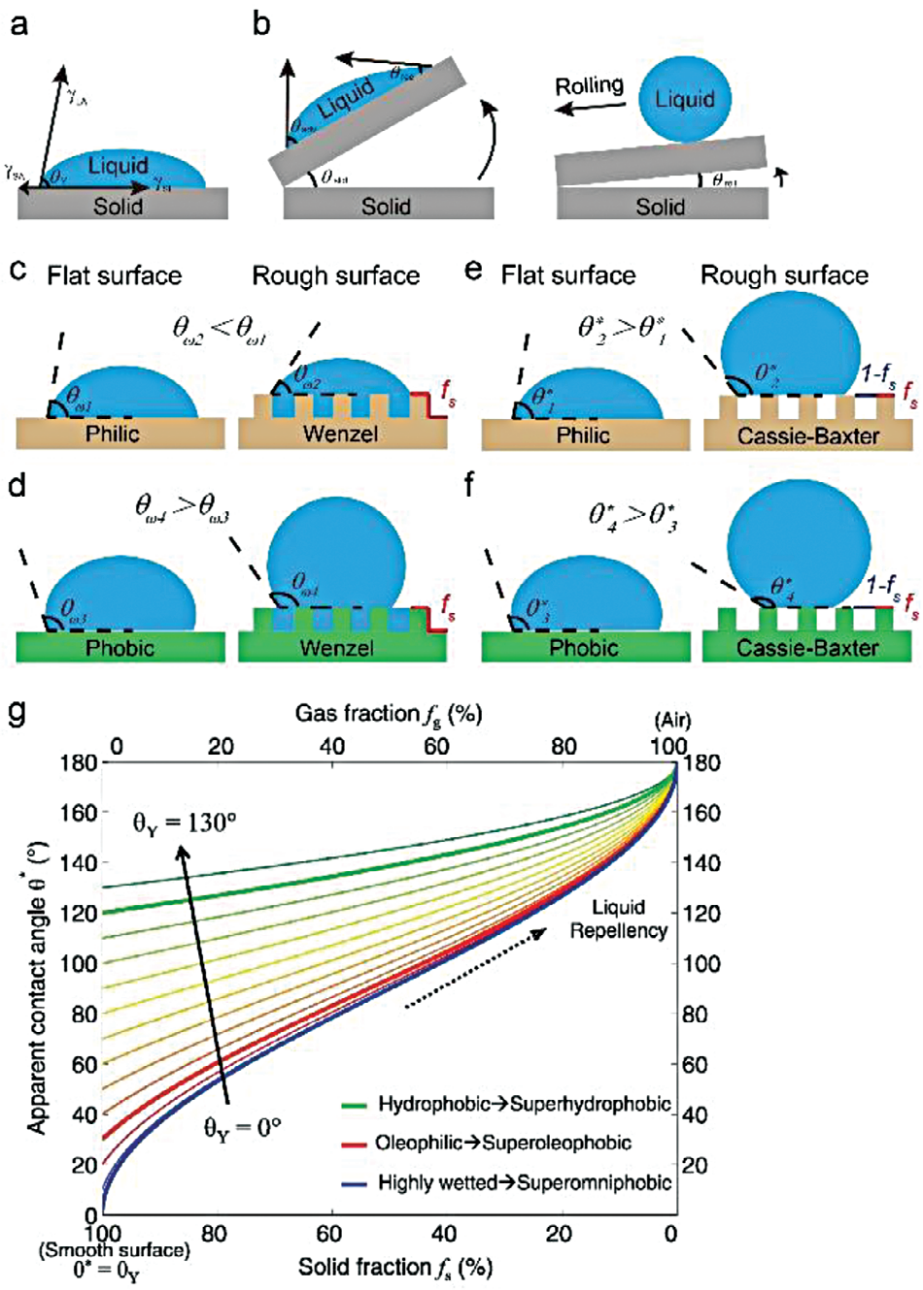
a) Young's contact Angle and its relation to surface energy. b) Diagram of advancing angle, receding angle and sliding/roll‐off angle. c–f) Schematic of Wenzel's model and Cassie‐Baxter's model respectively. g) Relationship between the apparent contact angle (***θ*** ^*^) and the liquid‐solid contact fraction (***f***) for an ideal Cassie‐Baxter state at different values of the static contact angle (***θ***_***Y***_). g) Reproduced with permission. Copyright 2014, AAAS.

If the solid‐liquid interface is Wenzel state, the greater the roughness (*r*) on the hydrophilic solid surface (θ_*Y*_<90°), the smaller θ_*w*_, the more wettable it will be; the greater the roughness (*r*) on the hydrophobic solid surface (θ_*Y*_>90°), the larger θ_*W*_, means the more non‐wettable (**Figure** **7**a,b).

In order to further adapt to complex situations in practical applications, Cassie and Baxter proposed a wetting model suitable for heterogeneous surfaces made of various components with different solid‐liquid (γ_*i*,*SL*_) and solid‐air (γ_*i*,*SA*_) interfacial tension.^[^ ^58^ ^]^ The fraction of each component in the surface is *f_i_*, wherein *f*_1_ + *f*_2_ + ⋅⋅⋅ +  *f_n_* =  1.The apparent contact angle (θ*) is shown by the following formula:

(17) $$\cos\theta^{*}=\frac{1}{\gamma_{LA}}\sum_{1}^{n}f_{i}\left(\gamma_{i,SA}-\gamma_{i,SL}\right)=\sum_{1}^{n}f_{i}\cos\theta_{Yi}$$

In particular, if the surface is only composed of a solid phase (its fraction is *f_s_*) and the air phase (its fraction is 1 − *f_s_*), which means air could be trapped below a droplet within the pore of the rough surface (Figure 7c,d), thus the θ* can be obtained by only introducing the static contact angle, θ_*Y*_:^[^ ^59^ ^]^

(18) $$\cos\theta^{*}=f_{s}\;\cos\theta_{Y}+f_{s}-1$$

As shown in Figure 7e, two significant conclusions could be drawn. First, the apparent contact angle (θ*) of a rough surface can be improved by increasing the static contact angle (θ_*Y*_) of the solid material, which can be achieved through chemical modifications that decrease the solid surface energy. Second, the θ* increases as the solid fraction (*f_s_*) decreases, which can be accomplished by increasing the surface roughness to reduce the solid‐liquid contact area. Furthermore, the roll‐off angle represents a pivotal parameter for the assessment of super‐liquid‐repellent surfaces. It can be intuitively defined as the angle of tilt at which a solid surface reaches a critical state just before a droplet commences rolling. The roll‐off angle is influenced by a myriad of contributing factors, including but not limited to solid surface energy, surface roughness, liquid surface tension, viscosity, and density, among others.^[^ ^56^ ^]^ In broad terms, a lower solid surface energy and a smaller solid‐liquid contact area also result in a smaller roll‐off angle.^[^ ^43c^ ^]^ Therefore, “micro‐ and/or nanoscale rough structures + low surface energy chemistry” is currently the general strategy for constructing super‐liquid‐repellent surfaces. The common materials used to decrease the surface energy are listed and categorized in **Table** **1**.

**Table 1:** Summary of common materials used to decrease the surface energy.

Classification	Examples
Fluorosilane	Trichloro(1H,1H,2H,2H‐tridecafluoro‐n‐octyl)silane, 1H,1H,2H,2H‐perfluorodecyltrichlorosilane, 1H,1H,2H,2H‐perfluorododecyltrichlorosilane
Siloxane	Trimethoxy(methyl)silane, Tripropoxy‐silane
Fluorosiloxane	1H,1H,2H,2H‐perfluorodecyltriethoxysilane, (1H,1H,2H,2H‐Perfluorododecyl)tris(ethoxy)silane
Alkyl stearate	Calcium stearate, 2‐Ethylhexyl stearate
Polymer	Polydimethylsiloxane, Polytetrafluoroethylene

#### 3.3.2 Dust Particle Adhesion on Surfaces

**Figure 8:**
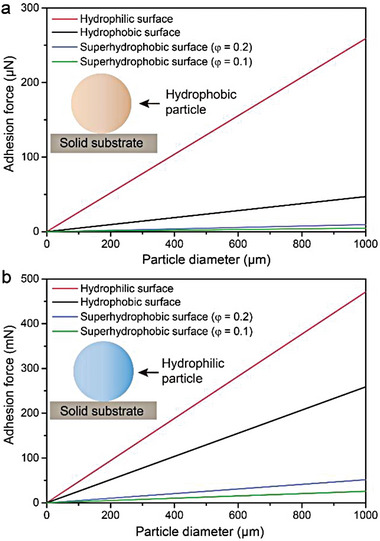
Adhesion force between particles and different solid surfaces. a) Hydrophobic (***γ***_**part**_ = 10 mN m^−1^) and b) hydrophilic (***γ***_**part**_ = 100 mN m^−1^) particle on surfaces as functions of surface energy and particle diameter.

For the self‐cleaning of liquid repellent surfaces, the natural effects such as gravity, rain droplet, moisture and wind are the major driving forces to remove dust, while the adhesion between particle pollutants and solid surfaces is the force to maintain particles adhering to the surface.^[^ ^54^, ^60^ ^]^ The key to successful self‐cleaning is ensuring that the driving force of particle separation is greater than the adhesion force. The JKR (Johnson‐Kendall‐Roberts) theory enables us to qualitatively and semi‐quantitatively assess the surface adhesion force (*F*_adh_) between a spherical particle and a flat surface:^[^ ^61^ ^]^

(19) $$F_{\mathrm{adh}}=\;\frac{3}{4}\pi D\varphi \left(\gamma_{\mathrm{surf}}+\gamma_{\mathrm{part}}-\gamma_{\mathrm{sp}}\right)$$

where *D* is the particle diameter, *φ* is the ratio of the actual to the apparent contact area of the particle. γ_surf_ is the surface energy of the substrate, γ_part_ is the surface energy of the particle. γ_sp_ is the interfacial energy between the substrate and particle, which can be neglected in this case. We estimated the γ_surf_ of the hydrophobic surface (fluorinated flat surface) as 10 mN m^−1^ and γ_surf_ of the hydrophilic surface as 100 mN m^−1^, φ are estimated as 0.1 and 0.2 for superhydrophobic surfaces respectively (0.1 means the better liquid repellency).^[^ ^62^ ^]^ As shown in (**Figure** **8**a,b), the adhesion between hydrophobic/hydrophilic particles and solid surfaces with different wetting properties were estimated.

In brief, regardless of the nature of the pollutant particles, the lower the solid surface energy, the smaller the contact fraction with particles (i.e., the greater the surface roughness), and the lower the adhesion. This is consistent with the design principle of the high‐performance liquid repellent surfaces.

#### 3.3.3 Dust Removal of Self‐Cleaning Liquid Repellent Surfaces

**Figure 9:**
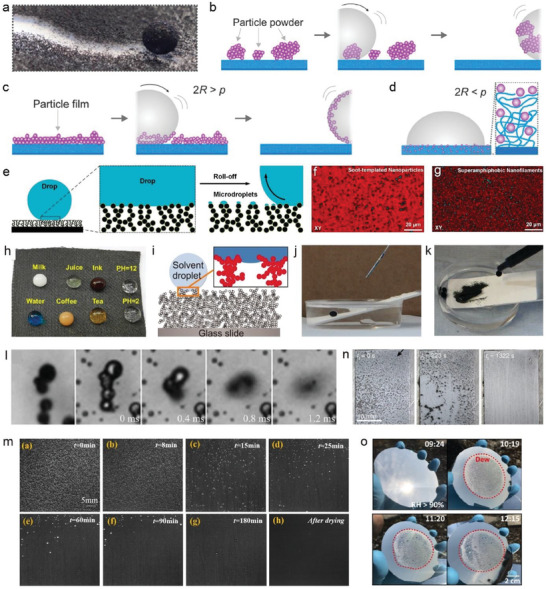
Dust removal of self‐cleaning liquid repellent surfaces. a) Photograph of a water droplet cleaning a superhydrophobic surface covered by pollutants. b,c) Schematic illustration of the self‐cleaning process of b) hydrophobic particle powders and c) hydrophilic particles (*p* < 2*R*) deposited from ethanol solution by a water drop on a superhydrophobic surface. d) Particles with a diameter smaller than the pore (*p* > 2*R*) can penetrate the surface micro‐nano structure, affecting wetting performance. e) Schematic of a drop rolling over a nanoparticle‐based superhydrophobic surface and microdroplets residue. f,g) Confocal images of the microdroplets residue on different superhydrophobic surfaces. h) Photograph of various liquid droplets on a superhydrophobic surface. i) Illustration of a solvent drop deposited a superamphiphobic surface. j) Water droplet was repelled by the TiO_2_ nanoparticles‐based superhydrophobic surface when immersed in oil (hexadecane). k) The dust on the superhydrophobic surface was cleaned by a water droplet in oil. l) Microscopic images showing the self‐cleaning process by self‐propelled jumping condensate. m) The process of soiling removal by condensate on superhydrophobic surface. n) Schematic showing the moisture‐driven superhydrophobic self‐cleaning. o) Qualitative images of dew harvesting on superhydrophobic surfaces under solar radiation. a–d) Reproduced with permission.^[^ ^62a^ ^]^ Copyright 2020, AAAS. e–g) Reproduced with permission.^[^ ^64^ ^]^ Copyright 2020. American Chemical Society. h) Reproduced with permission. Copyright 2021, American Chemical Society. i) Reproduced with permission.^[^ ^67^ ^]^ Copyright 2012, AAAS. j,k) Reproduced with permission.^[^ ^54c^ ^]^ Copyright 2015, AAAS. l) Reproduced with permission.^[^ ^54d^ ^]^ Copyright 2013, m) Reproduced with permission.^[^ ^70^ ^]^ Copyright 2021, Elsevier. n) Reproduced with permission.^[^ ^62b^ ^]^ Copyright 2020, Springer Nature. o) Reproduced with permission.^[^ ^71^ ^]^ Copyright 2021, AAAS.

Self‐cleaning of liquid‐repellent surfaces has garnered immense interest due to its diverse applications.^[^ ^63^ ^]^ The most prevalent technique for self‐cleaning entails removing contaminants through rolling water droplets on the hydrophobic nanotips of rough topography (**Figure** **9**a).^[^ ^62a^ ^]^ While this is a macroscopic phenomenon, understanding the self‐cleaning process from a microscopic perspective is pivotal to design high‐performance self‐cleaning surfaces.

To this end, Geyer et al. systematically studied the relationship between pollutant particle size and the pore diameter of surface microstructures.^[^ ^62a^ ^]^ Their findings demonstrated that hydrophobic micro‐ and nanoparticles on nanofilament‐coated superhydrophobic surfaces could be effectively removed by droplets without impacting superhydrophobicity (Figure 9b). However, hydrophilic particles dispersed in ethanol, representing severe pollution, impose higher requirements for liquid‐repellent surfaces. When hydrophilic particle size (*p*) is smaller than pore size (2R), particles could enter micropores of the coating, rendering superhydrophobicity ineffective even after washing, as pollutant nanoparticles remain within the microstructure (Figure 9c,d). Besides, Wong et al. noted that that the cleaning liquid could cause residual droplets after dust removal on superhydrophobic surfaces (Figure 8e).^[^ ^64^ ^]^ These micro‐droplets and micro‐pollutants are only pinned to the surface nanostructure's top and do not impact the Cassie‐Baxter state (Figure 9f,g). Moreover, superhydrophobic surfaces exhibit durable self‐cleaning performance when applied to pure volatile liquids, such as water, for extended periods. However, caution should be exercised when using non‐volatile liquids due to the damage to the wettability of liquid repellent surfaces caused by frequent use.

In addition to water, there are numerous other liquids with low surface energy or high viscosity in real life that are easy to wet solid surfaces, which is also a major challenge for self‐cleaning. A surface that is both superhydrophobic and superoleophobic can significantly expand the range of cleanable solvents, thus holding great practical significance.^[^ ^65^ ^]^ For instance, Wang et al. reported a MXene/Ni chain/ZnO array cotton fabric that delivered durable self‐cleaning performance.^[^ ^66^ ^]^ Common liquids with varying surface energies and viscosities, such as water, milk, coffee, juice, and other acid and alkali solutions, formed spherical droplets on the fabric surface and did not penetrate the surface (Figure 9h). Additionally, Deng et al. developed a transparent superamphiphobic surface using a candle‐soot template method, which exhibited a contact angle of over 150° toward water (γ = 72.1 mN m^−2^), diiodiomethane (γ = 50.9 mN m^−2^), ethylene glycol (γ = 47.3 mN m^−2^), peanut oil (γ = 34.5 mN m^−2^), olive oil (γ = 32.0 mN m^−2^), and even hexadecane (γ = 27.5 mN m^−2^) (Figure 9i).^[^ ^67^ ^]^ The polluted superamphiphobic coatings could be effectively rinsed by water and hexadecane, with both solvent droplets wrapping around the sand and rolling off the surface. Interestingly, Lu et al. created a paint‐like suspension of perfluorosilane‐modified TiO_2_ nanoparticles, which maintained excellent self‐cleaning performance even after being immersed in oil ((Figure 9j,k).^[^ ^54c^ ^]^ This significantly expanded the application range of self‐cleaning surfaces, such as in lubricating bearings, gears, and other industrial scenarios. In comparison to rain, alternative means of surface water generation, such as dew, condensate water, and morning fog, offer distinct advantages when it comes to facilitating self‐cleaning processes.^[^ ^68^ ^]^ These approaches are inherently gentler, making them particularly well‐suited for delicate super‐liquid‐repellent surfaces. In contrast, rainfall often brings about intense precipitation, accompanied by gusty winds and the potential for scouring effects, which can prove detrimental to fragile micro‐ and nano‐surface structures. Moreover, dew, fog, and similar phenomena tend to manifest continuously during the night and early morning, generally in the form of smaller droplets. This characteristic enables frequent and uninterrupted cleaning, rendering it easier to dislodge and remove minute contaminants.^[^ ^69^ ^]^ Rainfall, on the other hand, is irregular and largely dependent on prevailing weather conditions, making it less effective at small cleansing particles from surfaces. Wisdom et al and Yuan et al exhibited the whole process of self‐cleaning by condensate droplets on superhydrophobic surfaces, showcasing their ability to fully remove contaminants (Figure 9l,m).^[^ ^54^, ^70^ ^]^ Wang et al. also demonstrated the potential for self‐cleaning on armor superhydrophobic surfaces in fog (Figure 9n).^[^ ^62b^ ^]^ In a 1322 s continuous fog environment, the dust on the superhydrophobic surface was completely removed (Figure 9o,p). Excitingly, PDRC technology itself has the potential to collect dew from the environment throughout the day via condensation, enabling passive dew‐based self‐cleaning in practical applications. For example, Haechler et al reported that at relative humidity > 90%, a superhydrophobic radiative cooling system could harvest dew even under high solar irradiation, thus providing a sustainable water source for self‐cleaning (Figure 9q).^[^ ^71^ ^]^

#### 3.3.4 Photocatalysis‐Induced Self‐Cleaning

As an efficient, safe and environmental‐friendly purification technology, photocatalysis has been widely recognized by the international academic and industrial circles.^[^ ^72^ ^]^ Photocatalytic self‐cleaning is to utilize the oxidation‐reduction ability of photocatalyst to achieve the purpose of purifying pollutants. The commonly‐used photocatalysts include TiO_2_, zinc oxide (ZnO), tin oxide (SnO_2_), zirconia (ZrO_2_), cadmium sulfide (CdS) and other sulfide oxides as well as a small amount of silver salts.^[^ ^73^ ^]^ TiO_2_, due to its high catalytic activity, light corrosion resistance, and low toxicity, has become the most valuable photocatalyst.^[^ ^74^ ^]^ Unlike the self‐cleaning of liquid repellent surfaces, photocatalytic self‐cleaning technology is usually based on hydrophilicity/superhydrophilicity.^[^ ^75^ ^]^ On superhydrophilic surfaces, liquids are almost completely spread out, forming a uniform water film that isolates the adhesion and adsorption between the soiling and the solid surface.^[^ ^76^ ^]^ In the real world, wind and fog/rain are natural powers for photocatalysis‐induced self‐cleaning, helping to maintain surface cleanliness. Two successful examples of combining this technology with PDRC are the works of Wei et al in chapter 4.2.2^[^ ^36^ ^]^ and Zhou et al in chapter 4.2.4.^[^ ^77^ ^]^

#### 3.3.5 Laboratory Contamination Characterization Method for PDRC Materials

**Figure 10:**
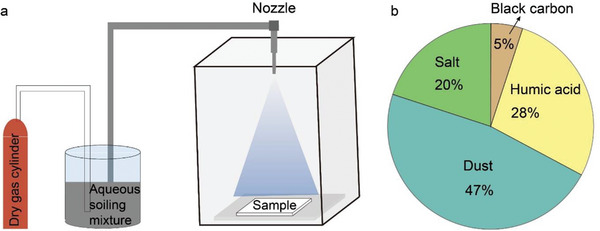
Laboratory contamination characterization method for PDRC materials. a) Composition and proportion of outdoor soiling used to simulate the three‐site average (Arizona, Ohio, and Florida). b) Schematic diagram of laboratory apparatus used for accelerated contamination. Adapted with permission.^[^ ^44^ ^]^ Copyright 2014, Elsevier.

As discussed in chapter 3.3.2, the characteristics of solid pollutants, such as shape, size, color, and the properties of cleaning liquids, such as surface energy and viscosity, can significantly affect the surface wettability to varying degrees, thus having different effects on the optical properties of PDRC materials. Therefore, it is imperative to establish unified and standardized testing methods to elucidate and compare the anti‐contamination performance of PDRC materials. However, most researchers have not adequately addressed this issue in their works, and the selection of soiling is relatively random, including but not limited to dust,^[^ ^35^, ^52^, ^77^, ^78^ ^]^ sand,^[^ ^25^, ^79^ ^]^ inorganic powder,^[^ ^52b^ ^]^ dyed liquids,^[^ ^19^, ^80^ ^]^ dirty water^[^ ^81^ ^]^ and mud,^[^ ^24^, ^51^ ^]^ among others. To address this gap, we recommend employing laboratory‐level soiling and weathering accelerated testing based on the widely‐accepted ASTM D7897‐18 standard.^[^ ^43b^ ^]^ This standard effectively simulates natural exposure equivalent to a period of three years. By incorporating a fixed proportion of four soiling mixtures, namely, soot, dust, particulate organic matter, and salts, this standard test allows for comprehensive evaluation of the changes in *R̅*_solar_ and ${\bar{\varepsilon }}_{\mathrm{LWIR}}$ resulting from air pollutants deposition in the real world (**Figure** **10**a). In Figure 10b, the soiling mixtures are evenly sprayed onto the sample surface through a spraying tank, followed by drying of the contaminated sample under an infrared heating lamp. Weathering steps before and after the soiling procedure replicate the effects of natural weathering of UV radiation and natural cleaning effects such as mist and precipitation. For specific quantitative parameters, readers are encouraged to refer to the ASTM D7897‐18 standard content. Implementing standardized soiling testing, as recommended, will greatly enhance the comparability and reliability of research findings concerning the anti‐contamination performance of PDRC materials.

### 3.4 Acid/Alkaline/Salty Solutions Corrosion

Acid rain, refers to atmospheric precipitation (e.g., rain, snow, frost) containing acidic substances, typically caused by factors such as the combustion of fossil fuels like coal, oil, and natural gas, as well as natural phenomena such as plant decay.^[^ ^82^ ^]^ Additionally, alkaline precipitation is also observed in specific environments, such as regions with alkaline soils or rocks, or in proximity to factories that emit alkaline waste gases.^[^ ^83^ ^]^ Therefore, acid/alkali corrosion represents a significant type of environmental aging that must be considered in polymer‐based PDRC materials. Similar to the photothermal aging mechanism mentioned above, acid corrosion aging results from the destruction of chemical bonds within polymer‐based materials, leading to changes in their physical and chemical properties. Specifically, chemical corrosion can cause a decline in toughness, strength, and other mechanical properties.^[^ ^84^ ^]^ Fracturing of surface chemical bonds can also lead to embrittlement and hardening of materials, which reduces wear resistance.^[^ ^85^ ^]^ Furthermore, acidic and alkaline environments can irreversibly damage and discolor the ultra‐white appearance of PDRC materials, thereby decreasing *R̅*_solar_ and making the sub‐ambient daytime cooling ineffective.^[^ ^79^, ^86^ ^]^ Inorganic compounds commonly‐used in PDRC, including metals and carbonates, can also be affected by acid and alkali corrosion. Such effects include chemical reactions, dissolution, and hydrolysis reactions, resulting in changes in properties such as color, texture, and structure.^[^ ^84^, ^87^ ^]^

**Figure 11:**
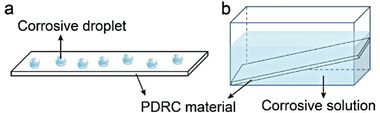
Schematic diagram of characterization methods for chemical corrosion. a) Corrosive droplet surface deposition method. b) Corrosive liquid immersion method.

To assess the chemical corrosion of PDRC materials, two regular characterization methods are commonly employed: corrosive liquid droplets surface deposition (**Figure** **11**a) and corrosive solution immersion (Figure 11b). While surface deposition is a convenient way to test optical performance and wettability, it may not accurately represent the effect of possible corrosivity on optical properties since it is limited to part areas of the surface. On the other hand, immersing PDRC materials in corrosive solutions for a certain time and then observing their optical performance and wettability is a more reliable way to evaluate chemical corrosion resistance. This characterization method has been adopted in most works on PDRC materials, including Fan et al.^[^ ^88^ ^]^ in 4.1.1.1, Wang et al.,^[^ ^86a^ ^]^ and Xue et al.,^[^ ^25a^ ^]^ Tian et al.,^[^ ^86b^ ^]^ Hu et al.^[^ ^79^ ^]^ in 4.2.3 and Huang et al,^[^ ^54b^ ^]^ in 4.3.2.

We summarized the test standards mentioned in chapter 3 in **Table** **2**, which will help the reader to reference efficiently and consult quickly.

**Table 2:** Test standards for different anti‐environmental aging properties of PDRC materials.

Aging categories	Test method	Ref.
Ultraviolet exposure	UV lamp irradiation	[14, 18, 22, 23]
	Sunlight exposure	[19, 22]
Thermal aging	In a thermal aging test chamber or on a heating platform	[19, 25, 52]
Flame retardancy	Flame burning method	[15, 24, 51]
Surface contamination	Liquid contamination method	[19, 35, 51, 80, 81]
	Dry powder contamination method	[35, 52, 77, 78, 79]
	Laboratory‐level soiling and weathering accelerated testing based on the ASTM D7897‐18 standard	[18c]
Chemical corrosion	Corrosive liquid droplets surface deposition	[43c]
	Corrosive solution immersion	[25, 79, 86, 88]

## 4 Designs and Fabrications of Anti‐Environmental Aging Passive Daytime Radiative Cooling Materials

In recent years, with the advancement of micro/nano manufacturing technology and the emergence of interdisciplinary research, the anti‐environmental aging performance of PDRC materials has become an important research field. By focusing on their anti‐contamination, physical and chemical durability, significant breakthroughs have been accomplished. However, achieving multiple durabilities in a single PDRC material is no easy feat, as different anti‐aging properties can sometimes interact or even hinder one another during the design process. Overcoming these challenges requires finding ways to stack new anti‐aging properties onto existing ones without sacrificing each other's performance. Moreover, the harmonious integration of PDRC and super‐wetting self‐cleaning technologies is an exciting frontier in surface science. The simplicity of the manufacturing process, environmental sustainability, and scalability are also crucial factors that determine the practical value of AEA‐PDRC materials. This chapter summarizes the recent developments in designing and fabricating AEA‐PDRC materials, including choosing environmental‐durable raw materials, designing special micro‐nano structures, optimizing surface chemistry and employing protective layers with environmental‐aging resistance.

### 4.1 Choosing Environmental‐Durable Raw Material

As discussed above, endowing PDRC materials with environmental aging resistance is the main focus of scientific research in the present day. To resist UV exposure, high temperature aging and flame burning, it is almost intuitive to start with the selection of raw material, because UV and thermal durability depends more on the physical and chemical properties of the raw material itself, and is not closely related to the derived structure. We discuss these strategies as follows.

#### 4.1.1 Employing Raw Materials with Stable Chemical Bonds

The purpose of using chemical bond stabilized raw materials is to obtain UV and thermal durability, which is the basis for further structural design to achieve anti‐contamination performance (to be discussed in detail in the chapter 4.2), requiring we understand from the molecular level. On the one hand, achieving excellent physical and chemical stability necessitates several fundamental characteristics. First, a simple and stable molecular structure is essential, without any chemical bonds that are prone to degradation. Moreover, the main chain should be composed of inert groups without reactive active groups to avoid any unnecessary chemical reactions. In other words, the chemical bonds must have a high energy, strength, and quantity to maintain stability. On the other hand, for PDRC, raw materials typically should also possess optical properties such as extremely‐high refractive index, high thermal infrared emittance, and low solar absorption. Only raw materials that meet the above conditions simultaneously can be considered ideal candidates for establishing AEA‐PDRC materials. The optical data of corresponding materials are shown in **Table** **3**, and they are all excellent candidates for building high optical performance PDRC. Note that the *R̅*_solar_ and ${\bar{\varepsilon }}_{\mathrm{LWIR}}$ of PDRC materials are deeply affected by the derived structure and surface characteristics. Therefore, in this chapter, we can solely provide the intrinsic optical properties of the raw materials, and the structural design and surface modification will be illustrated explicitly in chapter 4.2. For *R̅*_solar_, we provide the refractive index of raw materials for PDRC. Generally, a substantial difference in refractive indices contributes to a higher overall *R̅*_solar_; For ${\bar{\varepsilon }}_{\mathrm{LWIR}}$, we provide the main functional groups of polymeric raw materials and chemical bonds of inorganic raw materials, accompanied by their corresponding vibrational regions. Typically, the high ${\bar{\varepsilon }}_{\mathrm{LWIR}}$ of polymers is enhanced by the collective stretching vibrations of functional groups whose emission/absorption peaks overlap the atmospheric transparency window.^[^ ^89^ ^]^ Similarly, inorganic materials owe their high ${\bar{\varepsilon }}_{\mathrm{LWIR}}$ to the infrared‐active phonon vibrations of chemical bonds, typically occurring in specific bands.^[^ ^11g^ ^]^

**Table 3:** Summary of optical data of raw materials for AEA‐PDRC.

Materials	Refractive index	Functional groups or chemical bonds	Vibration region
Poly(vinylidene fluoride‐cohexafluoropropene)	1.4	C─F, C─H	Fingerprint region (7–16 µm), X‐H stretching region (2.5–4 µm)
Polydimethylsiloxane	1.4	Si─O─Si, C─H	Fingerprint region (7–16 µm), Skeletal vibration region (16–25 µm), X‐H stretching region (2.5–4 µm)
Fluorosilane	1.3–1.5	C─F, Si─O	Fingerprint region (7–16 µm), X‐H stretching region (2.5–4 µm)
Siloxane	1.3–1.5	Si─O	Fingerprint region (7–16 µm)
Polyvinylidene fluoride	1.4	C─F, C─H	Fingerprint region (7–16 µm), X‐H stretching region (2.5–4 µm)
Polymethyl methacrylate	1.5	C═O, C─O, C─H	Fingerprint region (7–16 µm), Double‐bond region (5–7 µm), X‐H stretching region (2.5–4 µm)
Poly(tetrafluoroethylene)	1.4	C─F	Fingerprint region (7–16 µm)
Polymethylpenteneand	1.6	C─N, C─H	Fingerprint region (7–16 µm), X‐H stretching region (2.5–4 µm)
Polylactic acid	1.4‐1.5	C═O, C─O	Fingerprint region (7–16 µm), Double‐bond region (5–7 µm)
Cellulose	1.5	C═O, C─O	Fingerprint region (7–16 µm), Double‐bond region (5–7 µm)
Potassium titanate	1.5	Titanium‐oxygen, Potassium‐oxygen	Fingerprint region (7–16 µm), Skeletal vibration region (16–25 µm)
Barium sulfate	1.6‐1.7	Sulfur‐oxygen, Barium‐sulfur	Fingerprint region (7–16 µm)
Aluminum oxide	1.8	Oxygen‐aluminum, Oxygen‐aluminum‐oxygen	Fingerprint region (7–16 µm), Skeletal vibration region (16–25 µm), Double‐bond region (5–7 µm)
Silicon dioxide	1.6	Silicon‐oxygen	Fingerprint region (7–16 µm)
Rutile titanium dioxide	2.7	Oxygen‐titanium	Fingerprint region (7–16 µm), Skeletal vibration region (16–25 µm)
Melamine‐formaldehyde	1.5	C═N, C─N	Fingerprint region (7–16 µm), Double‐bond region (5–7 µm)

##### Organic Compound and Polymer

Organic compounds, especially polymers, are the most commonly‐used matrix materials for PDRC, mainly due to their good plasticity, processability, substrate adhesion, and large‐scale application. We only list some classic examples as a reference here, and there are still many suitable and high‐performance raw materials worth further exploration.

**Figure 12:**
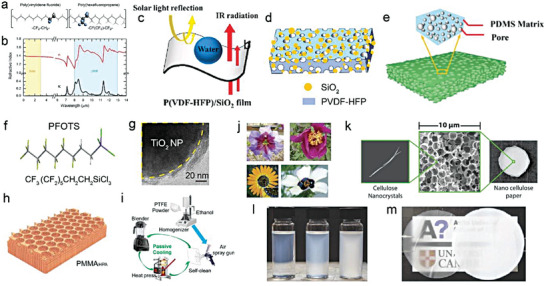
Employing raw materials with stable chemical bonds – organic compound and polymer. a) Structure diagram and b) spectral refractive index of P(VdF‐HFP). c) Schematic diagram of P(VdF‐HFP)/SiO_2_ film. d) Structure of P(VdF‐HFP)/SiO_2_ film. e) Schematic of porous PDMS film. f) Structure of perfluorooctyltrichlorosilane. g) TEM image showing the perfluorooctyltrichlorosilane grafted on the surface of TiO_2_ particle. h) Schematic of porous PMMA film. i) Preparation of PTFE‐based PDRC film. j) Structural colors based on natural components, such as cellulose, and lignin. k) Photo and SEM images of nano cellulose paper. l) Photo of the graded cellulose nanofibrils dispersions obtained by diluting to an equal concentration, showing the thinnest, medium, and coarsest fibrils from left to right. m) Photo of the compact cellulose nanofibril film (left) and 10 µm thick cellulose nanofibrils porous film (right) made from the coarsest fibrils. a,b) Reproduced with permission.^[^ ^19a^ ^]^ Copyright 2018, AAAS. c,d) Reproduced with permission.^[^ ^25a^ ^]^ Copyright 2022, Elsevier. e) Reproduced with permission.^[^ ^88^ ^]^ Copyright 2022. f,g) Reproduced with permission.^[^ ^18c^ ^]^ Copyright 2022, Springer Nature. h) Reproduced with permission.^[^ ^19c^ ^]^ Copyright 2021, Springer Nature. i) Reproduced with permission.^[^ ^86b^ ^]^ Copyright 2021, American Chemical Society. j) Reproduced with permission.^[^ ^97b^ ^]^ Copyright 2021, Springer Nature. k) Reproduced with permission.^[^ ^99^ ^]^ Copyright 2021, American Chemical Society. l,m) Reproduced with permission.^[^ ^100^ ^]^ Copyright 2021, Wiley‐VCH.

Poly(vinylidene fluoride‐cohexafluoropropene) (P(VdF‐HFP)) is one of the representative materials for AEA‐PDRC, attributing to its low intrinsic absorption in the solar spectrum, particularly in the ultraviolet region, and the strong radiation within the atmospheric transparent window (**Figure** **12**a,b).^[^ ^19^, ^25^, ^79^, ^90^ ^]^ The large difference in refractive index between PVdF‐HFP and air allows for effective scattering of sunlight, which results in high *R̅*_solar_. Additionally, the stable fluorine and carbon‐fluorine (C‐F) bonds exhibit high chemical inertness and resistance to external chemical attack. Furthermore, the high melting point (above 150 °C) of PVdF‐HFP confers good heat resistance and antioxidant properties, ensuring its physical and chemical stability. Previous studies have confirmed the powerful advantages of P(VdF‐HFP) as a raw material for AEA‐PDRC. Mandel et al. placed the porous (P(VdF‐HFP)_HP_) coating outdoors for one month and observed no significant decrease in optical performance, which is mainly attributed to the excellent UV and thermal stability, chemical resistance and high mechanical strength (exceeding that of traditional polyvinylidene fluoride) of (P(VdF‐HFP)_HP_).^[^ ^19a^ ^]^ Even after accelerated aging in a high temperature and humidity environment for 14 days, the P(VdF‐HFP)_HP_) coating could still maintain a high *R̅*_solar_ and ${\bar{\varepsilon }}_{\mathrm{LWIR}}$ of more than 0.9. Xue et al. fabricated a P(VdF‐HFP)/SiO_2_ composite film (Figure 12c,d) and confirmed that it could still maintain performance after experiencing immersion in solutions with different PH for up to 72 h, 200 h of UV irradiation and 60 cycles of sand‐paper abrasion.^[^ ^25a^ ^]^ Moreover, Hu et al. also also demonstrated that a P(VdF‐HFP) fiber‐based superhydrophobic PDRC film has superior UV resistance, maintaining unchanged optical and wetting properties even after 10 days of UV exposure.^[^ ^79^ ^]^

Polydimethylsiloxane (PDMS) is also frequently utilized to construct AEA‐PDRC materials, owing to following several aspects. First, PDMS is rich in high bond energy's, showing strong resistance to UV and thermal aging and chemical corrosion.^[^ ^91^ ^]^ Additionally, PDMS molecules contain a plethora of methyl groups, which form large angles with the silicon‐oxygen (Si‐O) bonds generated by oxygen atoms. Consequently, the molecular chain structure assumes an irregular shape, leading to slower chain segment movement. This, in turn, makes the molecules less susceptible to depolymerization reactions.^[^ ^92^ ^]^ Fan et al. conducted UV irradiation and acid and alkali solution immersion experiments upon the superhydrophobic porous PDMS cooling film (Figure 12e), respectively.^[^ ^88^ ^]^ The results showed that the fluctuation of the sub‐ambient temperature drop of the after‐testing film did not exceed 10%, demonstrating the excellent environmental aging resistance of PDMS. Zhao et al. proposed a switchable PDMS film via mechanical force to achieve white/transparent conversion.^[^ ^93^ ^]^ The film stayed unchanged optical performance after severe environmental aging, such as rain, snow, UV radiation, high (200°C) and ultra‐low (−196 °C) temperatures.

In addition, silanes, such as fluorosilane and siloxane, widely‐used for surface chemical modification, are known for their excellent stability, owing to the unique chemical properties of fluorine and silicon atoms.^[^ ^43^, ^63^, ^92^, ^94^ ^]^ This is particularly important, as most anti‐contamination liquid‐repellent PDRC materials incorporate the use of fluorosilane chemicals. Furthermore, the collective stretching vibrations from C─F and Si─O bonds and others from silanes within the atmospheric transparency window effectively improve thermal infrared emittance.^[^ ^18^, ^95^ ^]^ Wang et al. demonstrated that, due to the protective properties of long‐chain fluorosilanes, the porous PMMA film modified with 1H,1H,2H,2H‐perfluorooctyltrichlorosilane remained unchanged in appearance, color and wettability even after 480 h of accelerated weathering treatment.^[^ ^19c^ ^]^ Li et al. developed a super cooling wood and the surface modification with fluorosilanes chemically bonded to the wood channel improved its durability against fire, UV exposure, and biological factors, while also providing self‐cleaning properties by effectively preventing the penetration of mist.^[^ ^16a^ ^]^ Besides, Song et al also proved that 1H,1H,2H,2H‐perfluorooctyltrichlorosilane‐coated TiO_2_ nanoparticles could withstand more than 1000 h of UV exposure (Figure 12f,g). Nevertheless, it is imperative to underscore that the manufacturing and laboratory processing of fluorosilicone chemicals entail the use of toxic solvents and procedures, thereby giving rise to a range of environmental concerns.^[^ ^96^ ^]^ For instance, the production and processing of fluorosilicone chemicals typically involve the employment of toluene, xylene, ether, and various other organic solvents as carriers and diluents. Regrettably, this practice inevitably results in the emission of volatile organic compounds during the evaporation process, which, in turn, poses adverse ramifications for both atmospheric quality and human health.^[^ ^96a^ ^]^ Moreover, the application of high‐temperature drying, sintering, or other heat‐intensive procedures in the production process may engender the release of noxious gases.^[^ ^96b^ ^]^

Last but not least, there are still many candidates with stable chemical bonds for AEA‐PDRC, such as polyvinylidene fluoride,^[^ ^52^, ^54^ ^]^ polymethyl methacrylate (Figure 12h),^[^ ^15^, ^19^ ^]^ poly(tetrafluoroethylene) (Figure 12i),^[^ ^14^, ^52^, ^86^ ^]^ polymethylpenteneand,^[^ ^11^, ^80^ ^]^ etc., which will be reflected explicitly in the following chapters. Specially, natural polymers such as cellulose and lignin, found in almost all plants, are the most abundant natural materials on earth (Figure 12j).^[^ ^97^ ^]^ Nano cellulose self‐assembly technology has been proved to be able to achieve the same or better optical properties as commercial scattering enhancers, thus avoiding the use of non‐degradable artificial polymers and inorganic compound substances.^[^ ^15^, ^98^ ^]^ Caixeiro et al. achieved four times the scattering strength of ordinary micro‐fiber paper by optimizing optics under the condition of using exclusively cellulose nanocrystals (Figure 12k).^[^ ^99^ ^]^ S. Toivonen et al. et al. reported a breakthrough in the field of light transmission control in membranes using pure cellulose nanofibrils. By utilizing the coarsest fibrils to construct structures only a few microns thick, the film was able to achieve an impressive level of bright whiteness, surpassing the scattering efficiency of traditional white materials (Figure 12l,m).^[^ ^100^ ^]^ Aside from that, due to its inherent excellent UV and thermal aging resistance, renewability, and biocompatibility, cellulose has become a promising and environmental‐friendly candidate for AEA‐PDRC raw materials.

##### Inorganic compound

**Figure 13:**
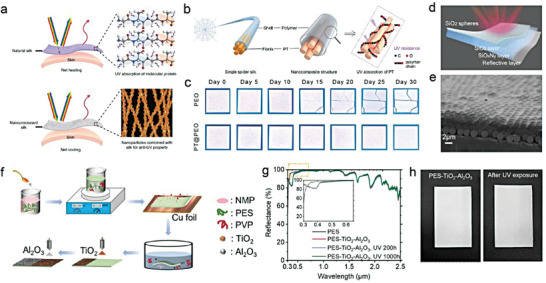
Employing raw materials with stable chemical bonds – inorganic compound. a) Schematic of natural silk and Al_2_O_3_ nanoparticles‐modified silk by enhancing UV reflectance. b) Spider silk‐like natural silk and nanocomposite doped with K_2_Ti_6_O_13_ designs and structures. c) Photographs of K_2_Ti_6_O_13_‐doped PEO film and pure PEO film after 30 days of outdoor exposure. d) Schematic and e) SEM image of three‐layer inorganic composite film. f) Schematic of the fabrication process of PES‐TiO_2_‐Al_2_O_3_ film. g) Reflectance spectra of PES‐TiO_2_‐Al_2_O_3_ film exposed to UV at different times. h) Appearance of the PES‐TiO_2_‐Al_2_O_3_ film before and after UV exposure. a) Reproduced with permission.^[^ ^102^ ^]^ Copyright 2021, Springer Nature. b,c) Reproduced with permission.^[^ ^22b^ ^]^ Copyright 2022, Wiley‐VCH. d,e) Reproduced with permission.^[^ ^23^ ^]^ Copyright 2022, Wiley‐VCH. f–h) Reproduced with permission.^[^ ^42^ ^]^ Copyright 2023, Wiley‐VCH.

Although several organic polymers with excellent performance are summarized above, the durability of organic compounds is still hard to compare with that of inorganic compound. This is mainly because the ionic and metallic bonds in inorganic compounds are usually more stable than the covalent bond in organic matter, which gives inorganic compounds higher durability in harsh environments.^[^ ^101^ ^]^ Zhu et al. employed a molecular bonding strategy using coupling agents to bind Al_2_O_3_ nanoparticles with diameter between 250 to 350 nm, which possessed strong scattering properties in the UV wavelength range, to silk. This innovative nano‐processing technology not only compensated for the inherent absorption of proteins in the UV band, but also offered the added benefit of protecting silk from UV aging (**Figure** **13**a). Yao et al. proposed a spider silk‐like composite polyethylene oxide (PEO) fibers film doping potassium titanate (K_2_Ti_6_O_13_) nanofibers that significantly enhanced the UV aging resistance and mechanical properties (Figure 13b).^[^ ^22b^ ^]^ The K_2_Ti_6_O_13_ nanofiber filler has three major advantages: high solar reflectance and thermal emittance, high mechanical strength, and the ability to mitigate UV light. Furthermore, the 720 h outdoor sunlight exposure aging test and the comprehensive environmental exposure test over 30 days and confirmed the excellent anti‐environmental aging performance (Figure 13c). Lin et al. creatively developed an all‐inorganic trilayer film, comprising of a monolayer of closely packed SiO_2_ microspheres, a SiOxNy film, and a Ag reflective film (Figure 13d,e).^[^ ^23^ ^]^ The SiOxNy layer and SiO_2_ microspheres could generate highly narrowband selective thermal emittance, while the SiOxNy layer allowed sunlight to pass through and be reflected by the underlying Ag layer, resulting in high *R̅*_solar_. Importantly, this all‐inorganic material without any fragile organic components successfully avoided environmental aging and degradation under sunlight, while its dense internal structure prevented the infiltration of external liquid. The 3 months UV radiation test and the 3 weeks immersion test have proved its superb UV resistance and water immersion resistance. Besides, rutile TiO_2_ is widely regarded as the most ideal white pigment due to its high UV and thermal durability, non‐toxicity, high melting point, and the incomparable whiteness.^[^ ^18^, ^73^ ^]^ Moreover, TiO_2_ nanoparticles are also strong light scatters, commonly‐used as UV mitigation component, commercially available and cost effective.^[^ ^18c^ ^]^ On this basis, our group proposed a polymer binder‐free strategy of packing hydrophobic TiO_2_ nanoparticles to establish an anti‐aging cooling paint (AACP).^[^ ^18c^ ^]^ Even after a 1000 h accelerated UV exposure test equivalent to 1 year of Florida natural sunshine, the optical properties and wettability of AACP coating have not been significantly affected, which could be attributed to the UV and thermal stability of TiO_2_ and the strong C‐F bonds in 1H,1H,2H,2H‐perfluorooctyltrichlorosilane. Li et al. confirmed that adding a TiO_2_ layer and an Al_2_O_3_ layer on a hierarchically porous PES film could significantly improve its UV aging resistance (Figure 13f), especially the top Al_2_O_3_ layer could compensate for the UV absorption problem of the TiO_2_ layer to maximize *R̅*_solar_ (Figure 13g).^[^ ^42^ ^]^ The ultra‐white appearance of PES‐TiO_2_‐Al_2_O_3_ composite film did not change after UV exposure testing (Figure 13h).

#### 4.1.2 Using Raw Materials with Flame Retardancy

**Figure 14:**
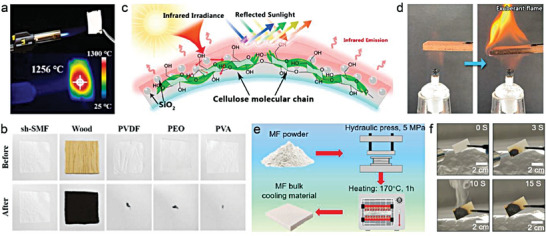
Using raw materials with flame retardancy. a) Photo and thermal infrared image of SMF film burning in flame. b) Photos of SMF film and comparison samples before and after flame combustion. c) Schematic of the cooling lignocellulosic bulk composed of SiO_2_ particles. d) The cooling lignocellulose bulk burned in a flame. e) Schematic of the manufacturing process of MF bulk. (f) The MF bulk shows excellent self‐extinguishing performance. a,b) Reproduced with permission.^[^ ^51^ ^]^ Copyright 2023, Elsevier. c,d) Reproduced with permission.^[^ ^22b^ ^]^ Copyright 2022, Wiley‐VCH. e,f) Reproduced with permission.^[^ ^24^ ^]^ Copyright 2021, Elsevier.

The flame retardancy of PDRC coatings or structural bulks is crucial for ensuring the safety of buildings, especially in case of emergencies.^[^ ^24^, ^51^ ^]^ The use of flame‐retardant raw materials is a general strategy for achieving qualified fire resistance. Compared to polymeric materials widely‐used in PDRC, inorganic materials have absolute advantages in ultra‐high temperature resistance, such as silica (SiO_2_), magnesium, aluminum, and other inorganic materials. Tsai et al. have fabricated a SiO_2_ metafibers (SMF) film via the electrospinning technique, with a high *R̅*_solar_ of 0.97 and ${\bar{\varepsilon }}_{8-13\mu m}$ of 0.9. The thermal stability of the SiO_2_ metafiber was evaluated by thermogravimetric analysis, which revealed no significant weight loss above 1100 °C, indicating excellent thermal resistance. Furthermore, the SMF film exhibited superior flame resistance, with no obvious changes in appearance observed after continuous flame combustion for 300 s, unlike other common polymer fiber‐based materials that rapidly turned to ashes in a matter of seconds (**Figure** **14**a,b). Crucially, SMF film retained the flexibility equivalent to polymer fibers, making them ideal for a wide range of applications. Chen et al. also demonstrated that incorporating SiO_2_ particles into cellulose‐based PDRC bulk could significantly improve the thermal stability and flame retardancy (Figure 14c,d).

Additionally, some thermosetting plastics with outstanding thermal stability could also be promising candidates for developing flame retardant PDRC materials. Melamine‐formaldehyde (MF) is a resin material that finds extensive application in various industries, including furniture and vehicle construction, owing to its exceptional thermal and mechanical stability. The unique feature of MF is its ability to occur curing and cross‐linking at temperatures exceeding 160 °C, resulting in increased hardness and flame retardancy without the need for any extra curing agents.^[^ ^24^, ^103^ ^]^ Based on this, Tian et al. developed a facile processing method to manufacture a porous MF bulk using MF particles.^[^ ^24^ ^]^ A wide size distribution of MF particles formed from particle coalescence and other interactions during the hot‐pressing process, resulting in strong sunlight scattering and excellent PDRC performance (Figure 14e). Besides, the MF bulk showed remarkable flame retardancy for building safety, igniting in a flame of 1430 °C and self‐extinguishing after 15 s (Figure 14f).

### 4.2 Designing Special Micro‐Nano Structures with Optimized Surface Chemistry

**Figure 15:**
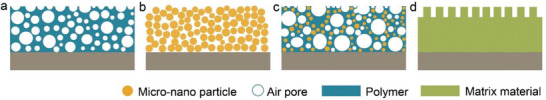
Schematic diagram of four special micro‐nano structural designs. a) Hierarchically porous structure. b) Micro‐nano pigments‐assembled dual‐scale structure. c) Particles/pigments‐combined porous structure. d) Surface‐patterned structure.

As discussed in chapter 2.2, *R̅*_solar_ is usually determined by the derived structure. In terms of anti‐environmental aging performance, apart from the inherent properties of the raw materials as we mentioned in the last chapter, the dual‐scale architecture and surface chemical modification are key elements in constructing liquid repellent surfaces, thereby endowing PDRC materials with the ability of repelling soiling. Therefore, designing special micro/nanostructures to improve optical performance and achieve liquid repellency simultaneously is also of interest. This chapter is the core for designing AEA‐PDRC materials, which can be mainly divided into four structural designs: hierarchically porous structure (**Figure** **15**a), micro‐nano pigments‐assembled dual‐scale structure (Figure 15b), particles/pigments‐combined porous structure (Figure 15c) and surface‐patterned structure (Figure 15d).

#### 4.2.1 Hierarchically Porous Structure

The air/solid interface at the micro‐nano scale has shown to effectively scatter light, with air voids of matching size to the solar wavelength resulting in high *R̅*_solar_. Various fabrication techniques, such as the phase‐inversion‐based method,^[^ ^15^, ^19^, ^104^ ^]^ sol‐gel method,^[^ ^105^ ^]^ and micro‐emulsion method,^[^ ^88^ ^]^ have been employed to produce polymeric materials with controllable porous structures. In addition, a well‐designed porous structure can trap air on the surface and lift droplets to realize the Cassie‐Baxter state, thereby achieving superhydrophobicity. Therefore, the intrinsic properties of raw materials and the overall structural design play crucial roles in determining the anti‐environmental aging properties. Accordingly, the air void‐based approach has emerged as a practical, versatile, and substrate‐independent strategy for developing AEA‐PDRC materials, showing great promise for commercial applications.

##### Porous Polymeric Coating/Film

In chapter 2.2.2, we have thoroughly demonstrated that porous structures, which are excellent light scatterers, serve as ideal models for PDRC in terms of optics. In recent years, material scientists have made strides in creating diversified porous materials that enable sub‐ambient cooling during the daytime. As we mentioned in 4.1, the utilization of raw materials that resist UV and thermal aging, chemical corrosion, and mechanical abrasion can directly produce AEA‐PDRC materials. Furthermore, chemical treatments and fine pore regulation can help create surfaces that are liquid‐repellent and resistant to surface contamination.

**Figure 16:**
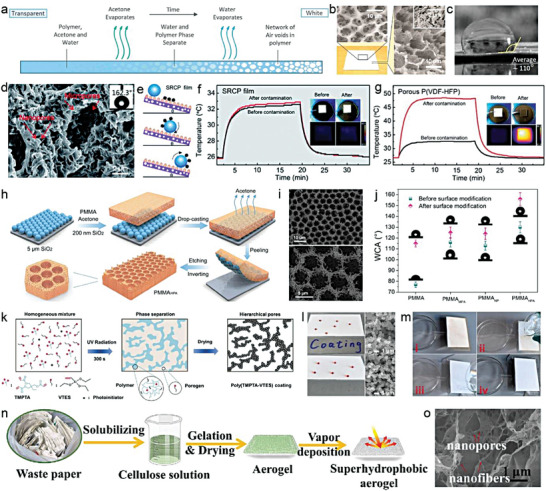
Porous polymeric coating/film. a) Schematic diagram of phase‐inversion‐based method for fabricating P(VdF‐HFP)_HP_ coating. b) SEM images showing top and cross‐section views of P(VdF‐HFP)_HP_. c) Photograph showing a water droplet placed on the P(VdF‐HFP)_HP_ coating. d) SEM image showing the surface structure of the SRCP film. The inset shows the water contact angle. e) Self‐cleaning process diagram of SRCP film. f,g) Temperature distribution of f) SRCP film and g) porous P(VdF‐HFP) film under simulated sunlight before and after being polluted by mud. h) Schematic illustration of the fabrication of PMMA_HPA_ with a hierarchically porous array. i) SEM images showing top views of PMMA_HPA_. j) Water contact angle variations of PMMA, PMMA_MPA_, PMMA_NP_, PMMA_HPA_ films after surface modification. k) Schematic illustration of photopolymerization‐induced phase separation method for fabricating the porous coating. l) Optical and m) SEM images of the poly (TMPTA‐VTES) coating before (top) and after (down) UV and moisture exposure for 50 days. m) Photographs of the self‐cleaning of the poly (TMPTA‐VTES) coating. (n) Schematic illustration of the preparation of waste paper‐based PDRC aerogel. o) Cross‐sectional SEM image of superhydrophobic cellulose aerogel cooler. a–c) Reproduced with permission.^[^ ^19a^ ^]^ Copyright 2018, AAAS. d–g) Reproduced with permission.^[^ ^22c^ ^]^ Copyright 2021, Royal Society of Chemistry. h–j) Reproduced with permission.^[^ ^19c^ ^]^ Copyright 2021, Springer Nature. k–m) Reproduced with permission.^[^ ^25b^ ^]^ Copyright 2021, American Chemical Society. n,o) Reproduced with permission.^[^ ^78^ ^]^ Copyright 2021, Elsevier.

As a classic example, Mandel et al. pioneered a facile, scalable and inexpensive phase‐inversion‐based process to fabricate a hierarchically porous P(VdF‐HFP) (P(VdF‐HFP)_HP_) coating (**Figure** **16**a).^[^ ^19a^ ^]^ Specifically, the precursor solution consisted of P(VdF HFP) (polymer), water (non‐solvent) and acetone (solvent). The wet coating naturally dried in air, and the rapid evaporation of acetone accelerated the phase separation between P(VdF‐HFP) and water, forming micro‐nanopores in the dry coating that effectively scatter sunlight and enhance ${\bar{\varepsilon }}_{8-13\mu m}$ (Figure 16b). The surface roughness caused by the hierarchical structure and low surface energy of P(VdF‐HFP) provided a θ* of 110° (Figure 16c). This work opened a new chapter in porous, paint‐based, AEA‐PDRC materials and brought excellent prospects. It garnered the attention of many material researchers, who continued to promote its development and inspired a lot of follow‐up works.

In order to further improve the contamination resistance of porous AEA‐PDRC materials, A promising approach is to finely tune micro‐nano porous structures to strengthen the surface roughness. Because as we can see, although the P(VdF‐HFP)_HP_ coating reached excellent hydrophobicity, the lack of surface roughness made it impossible to achieve superhydrophobicity. Excitingly, micro‐nano pores exhibit scale overlap and consistency in constructing high *R̅*_solar_ and liquid repellency. By accurately matching the distribution of pores and the wavelength of the solar spectrum to provide high sunlight scattering efficiency, while the surface dual‐scale composite structure formed by air voids could minimize liquid‐solid contact fraction and further decrease surface adhesion. Taking inspiration from a variety of bionics, Liu et al. improved this missing surface roughness by adding PDMS and using a dual‐component mixed solvent system, thereby creating a superhydrophobic radiative cooling porous (SRCP) coating via liquid templating technology.^[^ ^22c^ ^]^ The micro‐nano rough structure required to achieve superhydrophobicity was formed due to the competitive mass transfer diffusion process of two mixed polymers and the different volatilization mechanisms of two component mixed solvents. The derived SRCP coating contained abundant micropores with diameters from 2 to 10 µm, connected by multi‐scale 100–500 nm nanopores, creating an ideal dual‐scale rough surface to capture air pockets, where water droplets could completely rebound (Figure 16d,e). Besides, the self‐cleaning ability of the SRCP coating to protect and repair cooling performance is also important. The surface temperature of SRCP samples before and after contamination was basically unchanged (Figure 16f). In contrast, the hydrophobic porous P(VdF‐HFP) coating, representing the state‐of‐the‐art levels of optics, was heated up by 15.8 °C after contamination, rendering the cooling ineffective (Figure 16g). In another innovative work, Wang et al. reported a hierarchically porous PMMA film (PMMA_HPA_) for efficient PDRC.^[^ ^19c^ ^]^ As depicted in Figure 15h, A simple unidirectional friction assembly approach was used to prepare a tightly arranged hexagonal 5 µm SiO_2_ microspheres array. Then, the acetone dispersion composed of PMMA and 200 nm SiO_2_ nanospheres was infiltrated into the monolayer template. After the acetone volatilized, the regular and symmetrical micropores (≈4.6 µm in diameter) array and the random nanopores (≈250 nm in diameter) were achieved by etching SiO_2_ nanospheres and SiO_2_ microspheres template in aqueous hydrofluoric acid solution (Figure 16i). The surface modification of fluorosilane effectively improved the liquid repellency of PMMA_HPA_ film, and had almost no negative effect on the optical properties. In particular, compared with three different structural PMMA membranes (flat non‐pore PMMA, only nanopore PMMA_NP_ and only micropore PMMA_MPA_) modified by the same chemical, PMMA_HPA_ film was the only one that can accomplish superhydrophobicity, confirming the significant contribution of micro‐nano roughness raised by multi‐scale pores to liquid repellency (Figure 16j). Luo et al. reported a cheap and simple photopolymerization‐induced phase separation (PIPS) method to prepare porous poly (trimethylolpropane triacrylate‐vinyltriethoxysilane) coating (Figure 16k).^[^ ^25b^ ^]^ The continuous pores distribution from 200 nm to 8 µm resulted in strong Mie scattering and high *R̅*_solar_, while the micro‐nano roughness of the surface and low surface energy of the methyl and methylene groups led to superhydrophobicity and excellent self‐cleaning performance (Figure 16l,m).

Moreover, the use of environmentally‐friendly raw materials to build porous PDRC materials can greatly reduce secondary pollution. Yue et al. developed a porous cellulose‐based radiative cooler from waste paper, which integrated high optical performance, thermal insulation and superhydrophobicity (Figure 16n).^[^ ^78^ ^]^ The cooler contained abundant micro‐nano pores (porosity 91.3 ± 0.2%) and was coupled with a thin silane layer to transform its hydrophilicity into superhydrophobicity (Figure 16o). Similarly, Fan et al. employed a facile and eco‐friendly emulsion templating technology to create a superhydrophobic porous PDRC film.^[^ ^88^ ^]^ Intriguingly, prolonged mechanical stirring of water and incompatible PDMS resulted in the formation of a uniform and stable opalescent water‐in‐PDMS emulsion. Upon heating, the emulsion yielded a cured porous PDMS film, which could be further refined by a simple surface sanding method to increase surface roughness. Leveraging inherent low surface energy of PDMS, this approach enabled the achievement of remarkable superhydrophobicity.

##### Porous Structural Bulk

**Figure 17:**
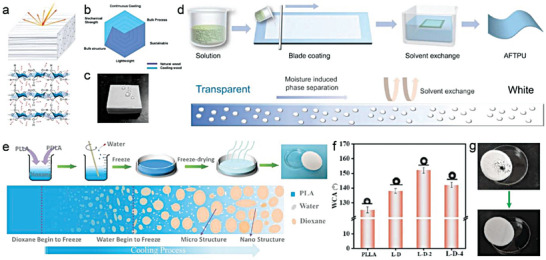
Porous structural bulk. a) Schematic showing the wood structure strongly scattering sunlight. b) Performance comparison between cooling wood and natural wood. c) Photograph showing water droplets placed on the cooling wood. d) Schematic of the fabrication process of AFTPU film. e) Fabrication process and schematic of the formation of micro‐nano structure of the SC‐PLA aerogel. f) Water contact angles of the PLA aerogels under various conditions. g) Self‐cleaning of the SC‐PLA aerogel with 2 ml water (L‐D‐2). a–c) Reproduced with permission.^[^ ^16a^ ^]^ Copyright 2019, AAAS. d) Reproduced with permission.^[^ ^107^ ^]^ Copyright 2022, Wiley‐VCH. e–g) Reproduced with permission.^[^ ^17c^ ^]^ Copyright 2022, Wiley‐VCH.

The combination of intrinsically durable porous materials and inert low surface energy chemical modification presents an ideal strategy for building AEA‐PDRC. This approach is not limited to surface materials, but also applies to bulk materials that can serve as mechanical supports. Li et al. reported on the development of a super cooling wood that could be cooled below ambient temperature under strong sunlight.^[^ ^16a^ ^]^ This cooling wood was created by densifying natural wood without lignin, leading to a large number of disordered pores that scatter sunlight strongly, generating high *R̅*_solar_. Enhanced infrared emission from collective stretching vibrations of cellulose fibers between 770 and 1250 cm^−1^ further contributed to high ${\bar{\varepsilon }}_{8-13\mu m}$ (**Figure** **17**a). As a high‐strength structural material, the cooling wood demonstrated excellent mechanical properties, including hardness, scratch hardness, bending strength, and compressive strength, which were several times higher than natural wood (Figure 17b). Surface modification enabled the super cooling wood to accomplish superhydrophobicity (Figure 17c).

In addition, expanding the use of air void‐based AEA‐PDRC materials to more advanced fields is crucial., an emerging technology with an extremely low density, has already found applications in aerospace, industrial production, and thermal insulation fields.^[^ ^17^, ^106^ ^]^ For instance, Leroy et al. have developed a porous polyethylene aerogel (PEA) with PDRC capability, which boasted a *R̅*_solar_ of up to 0.922 and a maximum ambient temperature daytime cooling power of 96 W m^−2^.^[^ ^17a^ ^]^ The thermal conductivity of PEA (28 ± 5 mW m^−1^ k^−1^) is nearly as low as that of air 26 mW m^−1^ k^−1^), making it highly suitable for use in water cooling systems and food preservation. Importantly, imparting environmental durability to aerogel could considerably enhance its active lifespan and reduce maintenance costs. Shan et al. developed an aerogel‐functionalized polyurethane (TPU) film (AFTPU film) for sub‐ambient cooling via the non‐solvent phase separation method (Figure 17d).^[^ ^107^ ^]^ The AFTPU film comprised a TPU substrate, with superhydrophobic SiO_2_ aerogel (SSA) particles serving as the functional units. The DMF solutions of TPU, containing varying concentrations of SSA particles, were applied to the solid substrate, resulting in phase‐separated films with strong light scattering, transitioning from a translucent state to an opaque white state upon exchange with water. The AFTPU film showed excellent optical performance and remarkable daytime cooling capacity, coupled with favorable hydrophobicity at a θ* of 135°. Moreover, AFTPU film can be shaped into various forms, thanks to its high flexibility and tailorability. Furthermore, by combining biomimetic hierarchical structures with stereocomposite (SC) crystals, Liu et al. have created a poly (Lactic Acid)‐based superhydrophobic PDRC (SC‐PLA) aerogel via a facile water‐assisted thermally induced phase separation (TIPS) method (Figure 16e).^[^ ^17c^ ^]^ The SC‐PLA aerogel possessed much richer multi‐scale air pores than pristine poly‐l‐lactic acid (PLLA) aerogels, enabling them to scatter sunlight more effectively. By increasing the water content in a specific range, the surface roughness of SC‐PLA aerogels increased significantly, forming a precise layered network composed of dual‐scale micro‐nano pores that achieved superhydrophobicity when the water with a volume of 2 ml (Figure 17f), demonstrating the self‐cleaning ability (Figure 17g).

#### 4.2.2 Pigments‐Assembled Dual‐Scale Structure

Pigment‐based paints and/or coatings are widely‐used in the field of high‐albedo roof materials due to their facile fabrication technique, low economic cost and eco‐friendly solvents.^[^ ^108^ ^]^ While the optical properties of the commercially available white paint‐based coatings are not enough to realize the PDRC, previous works have sufficiently demonstrated that the equivalent or even better optical performance than air void‐based PDRC materials can also be achieved by accurately matching of pigment size with the solar wavelength, adjusting pigment concentration, and utilizing pigments with high electron band gaps.^[^ ^18^, ^20^ ^]^ For example, Du et al. demonstrated that adding yttrium oxide (Y_2_O_3_) particles with a high refractive index, large bandgap, and high reflectance in the near‐infrared range, could significantly improve the *R̅*_solar_ of TiO_2_ particles‐embedded polymer binder white coating.^[^ ^18d^ ^]^ In another innovative study, Xue et al. developed a smart subambient radiative cooling coating using commercially TiO_2_ nanoparticles, glass microspheres and fluorescent pigments.^[^ ^18a^ ^]^ This coating leveraged mid‐infrared broadband radiation to deliver heat into outer space and carried out dynamic heat exchange within the atmospheric long‐wave radiation, strengthening the daytime cooling and suppressed the nighttime over‐cooling. Besides, Li et al. developed a BaSO_4_‐embedded acrylic coating with a large particle volume concentration of 60% and a wide particle size range of 398 ± 130 nm, reaching a nearly‐perfect *R̅*_solar_ of 0.981 and ${\bar{\varepsilon }}_{8-13\mu m}$ of 0.95.^[^ ^18b^ ^]^

**Figure 18:**
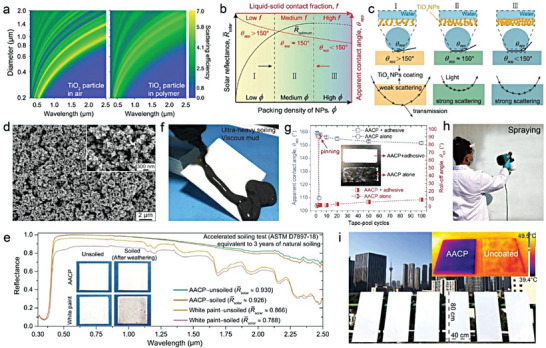
Pigments‐assembled dual‐scale structure. a) The scattering efficiency of a single TiO_2_ spherical particle as a function of particle diameter in different scattering media. b,c) Anticipated solar reflectance and apparent contact angle as a function of packing density of nanoparticles and the corresponding schematic diagrams. d) SEM image showing the AACP coating surface. e) Effect of soiling on the cooling performance in the real world of AACP and commercial white paint coatings. f) Photograph of AACP coating resistant to mud pollution. g) Improvement of robustness of AACP coating by adding primer adhesive. h) Demonstration of large‐scale spraying. i) Demonstration of AACP coating coated ceramic tiles. Reproduced with permission.^[^ ^18c^ ^]^ Copyright 2022, Springer Nature.

However, due to the lack of necessary surface roughness, the pigments‐embedded matrix structure is difficult to achieve surface liquid repellency. On this basis, our research group recently proposed a unique and straightforward strategy to create an anti‐aging cooling paint (AACP)‐based coating through solvent evaporation‐driven self‐assembly of pigment nanoparticles.^[^ ^18c^ ^]^ Specifically, AACP, which was an ethanol suspension of fluorosilane‐coated TiO_2_ nanoparticles, could be sprayed onto hard substrates to form a hierarchically dual‐scale pigments (diameter from 100 to 800 nm approximately) assembled coating after the solvent evaporation. Compared to the structure of TiO_2_ particles‐embedded polymer matrix, the large refractive index difference between TiO_2_ particles and air enabled the AACP coating to scatter sunlight more strongly, resulting in a higher *R̅*_solar_ (**Figure** **18**a). Additionally, compared to porous structure, the derived topology composed of randomly‐packed hydrophobic TiO_2_ nanoparticles have a lower solid‐liquid contact area, creating a dual‐scale micro‐nano surface roughness (Figure 18d), which could capture more air pockets, further reducing surface adhesion. The moderate packing density of nanoparticles effectively balanced and ensured superior optical performance and liquid repellency (Figure 18b,c). The *R̅*_solar_ of the tested AACP coating only decreased by 0.4% compared to the unaged ones after a simulated accelerated aging test equivalent to 3 years of outdoor natural soiling (Figure 18e). Furthermore, AACP coating also showed significant advantages in resisting heavy contamination, such as mud with high viscosity (Figure 18f). Moreover, adding commercial adhesives between the AACP coating and substrate greatly improved the robustness, which could resist external force damage such as high‐speed water jet impact, sand falling abrasion, and even tape‐peel (Figure 18g). This strategy of completely eliminating negative effects caused by polymer binders provided more ideas for the design of AEA‐PDRC materials. The integration of multiple strategies would greatly promote the development and application of AEA‐PDRC materials in the real world (Figure 18h,i).

In addition, many metal oxides, as pigments simultaneously, are also excellent photocatalysts and can provide additional self‐cleaning manners through photocatalytic oxidation reactions. Wei et al. developed a composite film composed of SiO_2_ nanospheres coated with a layer of TiO_2_ and ZnO nanorods (SiO_2_@TiO_2_/ZnO) for use in smart windows.^[^ ^36^ ^]^ The self‐cleaning performance of the coating was evaluated through degradation experiments involving methylene blue and the decomposition of stearic acid. The methylene blue on the surface of the coating gradually decomposed and became colorless under sunlight, with the decomposition products easily washed away by water. This excellent photocatalytic activity of SiO_2_@TiO_2_/ZnO film was mainly attributed to the wide band gap of both TiO_2_ and ZnO (greater than 3 eV), which prolonged the life of electron‐hole pairs generated by electronic excitation and improved the efficiency of photocatalytic oxidation reactions.

#### 4.2.3 Particles/Pigments‐Combined Porous Structure

**Figure 19:**
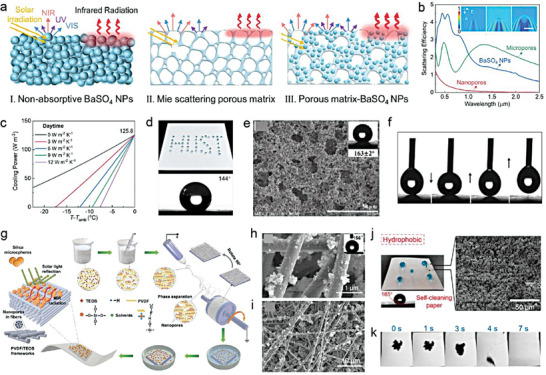
Particles/pigments‐combined porous structure. a) Schematic illustration of optical behaviors of three different structures. b) Scattering efficiency of BaSO_4_ nanoparticles and micro‐nano pores. c) Daytime net cooling power at different nonradiative heat exchange coefficients. d) Photographs of water droplets on the porous ethyl cellulose matrix−BaSO_4_ nanoparticles coating surface. e) SEM image of the P(VdF‐HFP)/SiO_2_ film. The inset shows the water contact angle. f) Water droplets detach from the P(VdF‐HFP)/SiO_2_ film surface. g) Microstructure schematic diagram and preparation process of SiO_2_ microspheres‐polyvinylidene fluoride/tetraethyl orthosilicate fiber composite film. h,i) SEM images of the PVdF‐HFP/SiO_2_ films. The inset in part h) is the water contact angle image. j) Surface‐wetting state and SEM image of microstructure of PTFE particles‐coated cellulose fiber composite film. k) Self‐cleaning process of PTFE particles‐coated cellulose fiber composite film. a–d) Reproduced with permission.^[^ ^107^ ^]^ Copyright 2022, American Chemical Society. e,f) Reproduced with permission.^[^ ^25a^ ^]^ Copyright 2022, Elsevier. g–i) Reproduced with permission.^[^ ^110^ ^]^ Copyright 2019, Wiley‐VCH. j,k) Reproduced with permission.^[^ ^79^ ^]^ Copyright 2022, American Chemical Society.

In chapters 4.2.1 and 4.2.2, we provide a detailed overview of the powerful advantages of air‐voids and pigments in constructing AEA‐PDRC materials. Although each strategy can improve the performance of PDRC, the complementary advantages of them have the potential to achieve even better optical properties. In a recent study, Liu et al. demonstrated a novel PDRC coating, incorporating BaSO_4_ nanoparticles‐embedded porous ethyl cellulose matrix, attained an exceptional *R̅*_solar_ of 0.986. This achievement is not only close to the theoretical limit, but also notably surpasses the *R̅*_solar_ values of pristine BaSO_4_ plates and porous ethyl cellulose films, which stood at 0.978 and 0.936, respectively (**Figure** **19**a).^[^ ^17b^ ^]^ This outstanding performance can be attributed to a combination of factors, including the high scattering efficiency of micro‐nano pores and collective behavior of Mie scattering from multi‐scale BaSO_4_ particles. Specifically, the micropores can strongly scatter the visible and near‐infrared light, and the nanopores further enhance the scattering effect in the UV‐visible region. BaSO_4_ particles can effectively scatter the entire solar spectrum, especially in the visible region (Figure 19b). These synergies provide broad spectrum scattering and high reflectance across the entire solar band. Aside from that, the stretching vibration of C‐O‐C within the atmospheric transparent window in ethyl cellulose and the high IR‐active phonon mode of BaSO_4_ also contribute to the promotion of ${\bar{\varepsilon }}_{8-13\mu m}$. This high optical performance is significant for strong cooling ability in the real environment, achieving a maximum net cooling power of 125.8 W m^−2^ at a heat balance state (Figure 19c). The coating also demonstrated excellent hydrophobicity (Figure 19d), mechanical stability, and recyclability.

In addition, the enhanced dual‐scale hierarchical structure formed by air voids and pigments can further increase surface roughness, synergistically strengthening the liquid repellency. Xue et al. successfully combined hydrophobic SiO_2_ nanoparticles with a classic hierarchically porous P(VdF‐HFP) film to significantly improve surface liquid repellency.^[^ ^25a^ ^]^ Compared to the unmodified porous P(VdF‐HFP) surface which had a θ* of 148°± 2°, the addition of 0.5‐2 µm SiO_2_ nanoparticles led to a dramatic increase in surface micro‐nano roughness, leading to an impressively high θ* of 163°± 2° and a near‐perfect roll‐off angle of 0.1 ± 0.1° (Figure 19e), The ultra‐low solid‐liquid adhesion facilitated droplet separation and roll‐off from the surface (Figure 19f). Similarly, Wang et al. developed a superhydrophobic porous PDRC film composed of ethylenepropylene‐diene copolymer (EPDM) and hydrophobic SiO_2_ particles, which showed great environmental durability in various tests, including mud contamination, immersion in acid and alkaline solutions, UV radiation, and mechanical abrasion.^[^ ^86a^ ^]^ Notably, an optimal amount of SiO_2_ particles accelerated the phase separation of EPDM to form pores, improving the *R̅*_solar_ to 0.96.

Furthermore, fiber‐based porous materials can be produced in a large scale by electrospinning, microfluidic spinning technology or electric spray method, which have garnered extensive attention owing to its straightforward manufacturing process and low cost.^[^ ^109^ ^]^ Wang et al. recently presented a novel flexible film, based on polyvinylidene fluoride/tetraethyl orthosilicate fiber and randomly distributed SiO_2_ microspheres, which showed excellent sub‐ambient daytime cooling performance (Figure 19g).^[^ ^110^ ^]^ The exceptional optical scattering from the porous nanofibers network, combined with the intrinsic phonon polaron resonance of SiO_2_ microspheres, led to enhanced *R̅*_solar_ and ${\bar{\varepsilon }}_{8-13\mu m}$. In a similar vein, Hu et al. developed a superhydrophobic flexible film by incorporating SiO_2_ nanoparticles into P(VdF‐HFP) fiber frameworks (Figure 19h,i).^[^ ^79^ ^]^ The low surface energy of SiO_2_ and the air cushion formed by the micro‐nano surface structure were the keys to achieving superhydrophobicity. In addition, poly(tetrafluoroethylene) (PTFE) particles with low surface energy are also an ideal choice for modifing fiber‐based PDRC materials. Tian et al. prepared a PTFE particles‐coated cellulose fiber composite film via a facile air‐spraying method, with a θ* of up to 165° (Figure 19j), showing superior self‐cleaning performance (Figure 19k).^[^ ^86b^ ^]^

#### 4.2.4 Surface‐Patterned Structure

Surface‐patterned materials are custom‐made structures with specific shapes and sizes that can be manufactured in a highly reproducible approach via photolithography.^[^ ^80^, ^111^ ^]^ Such microstructures can reflect sunlight effectively, a feature commonly observed in natural organisms. For example, the triangular structural fluffs found on the Sahara silver ant serve as a reflective layer that can reflect most of the sunlight.^[^ ^11a^ ^]^ Meanwhile, most liquid‐repellent surfaces are also constructed using surface micro‐patterns such as micropillar structures,^[^ ^112^ ^]^ single or double‐ structures,^[^ ^59^, ^113^ ^]^ and armor structures.^[^ ^62b^ ^]^ Therefore, a well‐designed surface‐patterned material can exhibit dual functions by taking advantage of the structural commonalities between photonic structural color and surface wettability.

**Figure 20:**
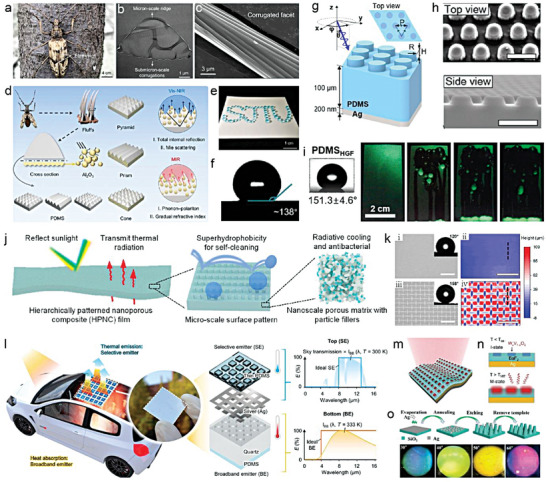
Surface‐patterned structure. a–c) Photographs and SEM images of fluffs on the forewings of N. gigas. d) Schematic diagram of the fluffs‐inspired composite films with photonic architectures. e) Photograph of water droplets on the Bio‐RC film. f) Water contact angle of Bio‐RC film. g) Schematic of a hexagonal‐array grating‐patterned PDMS film. h) Top view and side view SEM images of hexagonal‐array grating. i) Water contact angle of PDMS_HGF_ and dust removal via droplet impacts on the surface. j) Schematic diagram of the HPNC film. k) Optical images and confocal laser microscope images of surface morphology of flat patternless nano PE film and surface‐patterned nano PE film. l) Structure diagram and working principle diagram of JET. m) Schematic of the structure of TARC. n) Schematics of materials composition and optical transition mechanism of TARC. o) Fabrication process and structural color of the truncated microcone array. a–f) Reproduced with permission.^[^ ^80b^ ^]^ Copyright 2020, g–i) Reproduced with permission.^[^ ^111d^ ^]^ Copyright 2022, Elsevier. j,k) Reproduced with permission.^[^ ^77^ ^]^ Copyright 2023, American Chemical Society. l) Reproduced with permission.^[^ ^116^ ^]^ Copyright 2020, AAAS. m,n) Reproduced with permission.^[^ ^117^ ^]^ Copyright 2021, AAAS. o) Reproduced with permission.^[^ ^118^ ^]^ Copyright 2022, Wiley.

Learning from nature is a common strategy for designing surface microstructures. For instance, the surface of the forewing of the longicorn beetle is covered with fluff, with each fluff being a triangular hierarchical rough structure composed of one smooth facet and two corrugated rough facets that can resist color fading and show structural color characteristics of photonic crystals (**Figure** **20**a–c).^[^ ^114^ ^]^ This surface structure not only enhances solar reflectance but also radiates heat effectively into the universe, allowing longicorn beetles to survive in areas with extremely high temperatures.^[^ ^92^, ^115^ ^]^ Inspired by this, Zhang et al. employed photolithography to manufacture an inverted‐micropyramid shaped silicon wafer as a template (Figure 20d). A PDMS precursor solution with randomly distributed Al_2_O_3_ microspheres was spun onto the template. The cured biomimetic radiative cooling film (Bio‐RC film) showed excellent optical performance and cooling capacity, exhibiting an average temperature drop of 5.1 °C under the solar irradiance of 862 W m^−2^.^[^ ^80b^ ^]^ The top inverted‐micropyramid pattern of the Bio‐RC film led to a θ* of 138° (Figure 20e,f), much higher than the θ* of 114° observed on flat PDMS surface. The Bio‐RC film also demonstrated great flexibility and elasticity, retaining its functionality even after alternating torsion and hundreds of stretching cycles. In another example, Choi et al. fabricated a hexagonal‐array grating‐patterned PDMS film backed with a silver reflective layer (Figure 20g,h).^[^ ^111d^ ^]^ Although the primary aim of this grating pattern was to optimize the optical performance, it also synergistically improved surface liquid repellency (Figure 20i). This was mainly attributed to the grating pattern successfully trapped air under droplets, leading to the Cassie‐Baxter state. Zhou et al. reported a surface‐patterned bifunctional PDRC film based on a nano porous polyethylene matrix via a template‐molding technique (Figure 20j). On the one hand, the micropillar array pattern on the surface effectively reduced solid‐liquid adhesion, thereby inducing the realization of superhydrophobicity. Compared with flat hydrophobic nano polyethylene film, the introduction of micropillar array pattern increased the contact angle from 120° ± 2° to 158° ± 2°. On the other hand, ZnO nanoparticles, as photocatalysts, could absorb UV light and produce reactive oxygen species, resulting in bactericidal effects (Figure 20k). The soiling contamination and bacterial growth experiments further demonstrated the excellent self‐cleaning performance and anti‐bacterial function.

In addition, incorporating different coatings can not only improve the performance of surface‐patterned PDRC materials, but also provide them with more functions, thus broadening their range of applications. This includes, but is not limited to, cooling intensification layers,^[^ ^116^ ^]^ self‐adaptive switching layers,^[^ ^11^, ^117^ ^]^ and colorful layers that meet aesthetic needs.^[^ ^118^ ^]^ For example, Wang et al. designed a Janus emitter (JET) to further enhance the cooling in the enclosed spaces (Figure 20l).^[^ ^116^ ^]^ Briefly, the top layer of 4‐µm PDMS layer on Ag‐coated micro‐patterned quartz frame avoided environmental radiation heat through ideal narrowband emission, and the bottom layer of 10 µm thick PDMS layer carried out broadband emission to absorb accumulated heat in space. The experimental results also showed that JET is more effective in cooling enclosed spaces than unidirectional sky radiative materials. Addressing the challenge of excess cooling during winter, the development of PDRC technology with adaptive solar spectrum regulation capabilities is imperative to achieve the desired effect of “warm winters and cool summers.” Vanadium dioxide (VO_2_), a phase‐change material capable of high‐reflectance/high‐transmittance switching of infrared light depending on the external temperature, presents an ideal candidate for dynamic AEA‐PDRC raw materials.^[^ ^105a^ ^]^ Tang et al. designed a multi‐layer surface‐patterned temperature‐adaptive radiative coating (TARC) based on VO_2_, which could automatically adjust indoor temperature through roofs and walls (Figure 20m,n).^[^ ^117^ ^]^ Importantly, tungsten doping of 1.5% could effectively decrease the phase transition temperature from 67 °C to around room temperature (25 °C), thereby the ${\bar{\varepsilon }}_{8-13\mathrm{um}}$ of TARC could be switched from 0.2 to 0.9. Moreover, Ding et al. prepared a radiative cooler featuring a truncated SiO_2_ microcones array through self‐assembly and reactive ion etching (Figure 20o).^[^ ^118^ ^]^ Due to the scattering effect induced by the truncated microcone array, the cooler presented a continuous change from red to blue at different incident light angles, ensuring high optical performance while simultaneously satisfying aesthetic requirements.

### 4.3 Applying a Protective Layer with Environmental‐Aging Resistance

Multi‐layer films have been developed using a series of functional substances to enhance the performance of AEA‐PDRC.^[^ ^11^, ^14^, ^22^, ^35^, ^52^, ^95^ ^]^ These layers may include, but are not limited to, anti‐fouling liquid repellent layers,^[^ ^11^, ^35^, ^52^ ^]^ anti‐chemical corrosion air‐cushion layers.^[^ ^14^, ^22^, ^25^, ^54^, ^95^, ^111^ ^]^ and colorful layers that meet aesthetic needs. In fact, this strategy of overlapping functionality is a comprehensive application of the aforementioned raw material selection and structural design, and each single‐layer design can be found in the above discussion. However, integrating multiple functions into a single material remains a challenge due to the interdisciplinary nature of the field, and the fact that some functions may be mutually exclusive. Therefore, adding additional functions without compromising the optical ability is the most fundamental design principle. As materials science continues to evolve, researchers are exploring new ways to endow more functions to multi‐layer PDRC materials and push the boundaries of what is possible in the field.

#### 4.3.1 Adding a Liquid Repellent Top Layer

**Figure 21:**
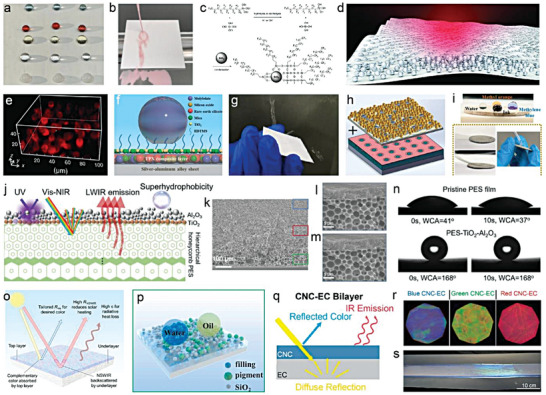
Multilayered PDRC films. a) Photograph of different solvent droplets on the superhydrophobic top layer. b) Photo of the superhydrophobic top layer resistant to dyed liquid. c) Chemical reactions during the preparation of the superhydrophobic top layer. d) Schematic diagram of polymer‐based hybrid metamaterial with resonant polar dielectric microspheres. e) 3D confocal microscope image of hybrid metamaterial. f) Schematic of the anti‐contamination of multi‐layer film. g) Photograph of the multi‐layer film impacted with the water jets. h) Schematic of the PTFE layer with a hierarchical micropattern and ceramic porous bottom layer with surface phonon‐polariton. i) Images of the bilayer film surface with different solvent droplets. j) Schematic of the structure of trilayer PES‐TiO_2_‐Al_2_O_3_ film. k) Cross‐sectional SEM image of the PES‐TiO_2_‐Al_2_O_3_ film. l,m) SEM image of the top area l) and the bottom area m) of the PES‐TiO_2_‐Al_2_O_3_ film. n) The θ* of the PES film and PES‐TiO_2_‐Al_2_O_3_ film. o) Optical schematic diagram of bilayer colorful PDRC film. p) Schematic of superhydrophobic multi‐layer colorful PDRC film. q) Optical schematic diagram of nanocrystals‐ethylcellulose bilayer film. r) Optical micrographs of the blue, green, and red cellulose nanocrystals‐ethylcellulose bilayer films and s) roll‐to‐roll process. a–c) Reproduced with permission.^[^ ^35b^ ^]^ Copyright 2022, Elsevier. d,e) Reproduced with permission.^[^ ^11g^ ^]^ Copyright 2017, AAAS. f,g) Reproduced with permission.^[^ ^80a^ ^]^ Copyright 2021, Elsevier. h,i) Reproduced with permission.^[^ ^52b^ ^]^ Copyright 2021, American Chemical Society. j–n) Reproduced with permission.^[^ ^42^ ^]^ Copyright 2023, Wiley‐VCH. o) Reproduced with permission.^[^ ^119^ ^]^ Copyright 2020, AAAS. p) Reproduced with permission.^[^ ^35a^ ^]^ Copyright 2021, Elsevier. q‐s) Reproduced with permission.^[^ ^15b^ ^]^ Copyright 2022, Wiley‐VCH.

As one of the most practical and intuitive design principles, directly utilizing of a liquid repellent top layer that is transparent to the solar spectrum to establish muti‐layer films has proved to be a great potential for anti‐contamination PDRC. Zhang et al. systematically investigated the effects of superhydrophobic finishes comprising hydrophobic SiO_2_ nanoparticles on the optical properties of pigment‐based PDRC coatings, demonstrating the feasibility of adding a ultra‐high sunlight‐transparent superhydrophobic top layer to PDRC materials.^[^ ^35b^ ^]^ The micro‐nano rough structure of the superhydrophobic layer, modeled on the Cassie‐Baxter model, enabled the trapping of air pockets on the surface, thereby enhancing its superhydrophobicity. Notably, even after being in contact with the coating surface for 24 h, water and hexadecane could still appear as spheres (**Figure** **21**a) and repelled any inorganic contaminants encountered while the surface was inclined at a 2° (Figure 21b), effectively completing the self‐cleaning process. Intriguingly, chemical reactions during the manufacturing of the superhydrophobic layer led to the formation of new bonds and stretching vibrations within the thermal infrared region (Figure 21c). As a result, the bilayer film's emittance in the atmospheric transparent window (8–14 µm) increased from 0.950 to 0.954, and the emittance in the wavelength range of 2.5–24 µm was also strengthened from 0.892 to 0.921.

At the micro and nano scale, the structures necessary for constructing liquid‐repellent surfaces overlap with and align with the requirements for optimal solar light scattering. Therefore, a well‐designed liquid‐repellent top layer not only maintains the optical performance of PDRC materials, but can also lead to synergistic enhancements in *R̅*_solar_. This represents a more advanced and desirable design strategy compared to applying a sunlight‐transparent layer. Zhai et al. have ingeniously proposed a metamaterial composed of a sunlight‐transparent polymer matrix encapsulating resonant polar dielectric microspheres (Figure 21d,e).^[^ ^11g^ ^]^ This metamaterial could achieve both high *R̅*_solar_ and high ${\bar{\varepsilon }}_{8-13\mu m}$ when backed with a thin silver reflective layer, possessing the PDRC capacity. On this basis, Tao et al. subsequently improved the anti‐contamination based on this metamaterial by modifying its surface.^[^ ^80a^ ^]^ They added SiO_2_, molybdate, mica, and rare earth silicate to the TPX matrix (Figure 21f), which broadened the infrared absorption peak across the entire atmospheric transparency window and effectively promoted selective emittance. They also deposited hydrophobic mica and TiO_2_ particles on the top layer, increasing surface roughness and resulting in great self‐cleaning performance (Figure 2g). Wang et al. drew inspiration from the multi‐scale fluffs of long‐horned beetles to create an organic‐inorganic bilayer film that combined PDRC and superhydrophobicity.^[^ ^52b^ ^]^ The top layer composed of PTFE with a “ridge” structure and randomly distributed nanoparticles, could not only strongly scattering sunlight (Figure 21h), but also provided large surface roughness to construct a superhydrophobic surface (Figure 21i).The micro‐nano porous ceramic bottom layer with lattice absorption assisted in enhancing ${\bar{\varepsilon }}_{8-13\mu m}$. The bilayer film maintained its robust superhydrophobicity after tape‐peeling, jet impacting, knife scraping, abrasive paper abrasion, and even 100 days exposure in the real‐world. Li et al. cleverly proposed an inorganic‐organic composite trilayer film for constructing a superhydrophobic and anti‐UV radiative cooler (Figure 21j,k).^[^ ^42^ ^]^ From bottom to top, the trilayer film was composed of porous polyether sulfone (Figure 21l), TiO_2_ nanoparticles UV absorption layer, and Al_2_O_3_ nanoparticles superhydrophobic top layer (Figure 21m). Compared to pristine PES film, the rough structure of nanoparticles on the top layer effectively increased the θ* from 41 ° to 168 ° (Figure 21n).

Furthermore, color, as an aesthetic demand, adding a colored top layer, making it possible to create colorful AEA‐PDRC materials.^[^ ^118^ ^]^ The mechanism of color generation can be divided into two categories, one is based on the selective light absorption of dyes, and the other is based on the selective light reflection, known as structural color. It is also worth mentioning that selectively absorbing light to obtain color is detrimental to cooling. For the first type, Chen et al. reported a bilayer colorful PDRC film with a thin colorant top layer and a typical PDRC under layer with air voids or pigments (Figure 21o).^[^ ^119^ ^]^ This design allowed near‐to‐short wavelength infrared light to pass through the colorant layer and be reflected by the PDRC under layer, resulting in high *R̅*_solar_. Tao et al. developed a colorful emitter by incorporating a high‐reflectance substrate, a selective emission film, and a sprayed pigments top layer (Figure 21p).^[^ ^35a^ ^]^ The top layer, consisting of perfluorooctyltriethoxysilane and hexafluoro‐butyl acrylate grafted SiO_2_ particles and pigments, provided diversified colors while maintaining excellent optical and wetting performance even after abrasion and UV exposure. Liu et al. introduced a colored bilayer coating with self‐cleaning properties and PDRC, comprising of zirconium dioxide (ZrO_2_) particles embedded in PVdF.^[^ ^52a^ ^]^ While maintaining high *R̅*_solar_ and UV resistance, the addition of colorants produced different colors of red, green, and blue, and provided surface roughness to construct a superhydrophobic surface. For the second type, Zhu et al. proposed an environmental friendly bilayer cellulose nanocrystal (CNC)‐based colorful film for PDRC (Figure 21q,r), which could be mass‐produced (Figure 21s).^[^ ^15b^ ^]^ The periodic helical photonic nanostructures of the CNC self‐assembled top layer could selectively reflect visible light, resulting in a range of colors depending on the pitch size. Besides, the sunlight penetrating the CNC layer in other wavelengths would be reflected by the ethyl cellulose bottom layer, thereby minimizing the absorption of sunlight and achieving high *R̅*_solar_. Therefore, future endeavors may focus on improving the environmental durability of sustainable colorful PDRC materials.

#### 4.3.2 Adding an Anti‐Chemical Corrosion Air‐Cushion Layer

Lastly, it is worth noting that the liquid repellent outer layer is also an effective strategy to protect PDRC materials from chemical corrosion. For instance, Hu et al. ascribed the remarkable chemical resistance of P(VdF‐HFP) fiber‐SiO_2_ particle composite film to two points: the cavitation structure of the liquid repellent layer that impeded the penetration of corrosive liquids and the inherent inertness of fluoride.^[^ ^79^ ^]^ Likewise, Huang et al demonstrated that the air cushion at the interface between the corrosive solution and the superhydrophobic surface protected the PDRC film from erosion by acid/alkali solutions. The superhydrophobic porous PDRC membrane based on PVdF/PDMS could maintain superhydrophobicity even after being immersed in solutions containing different PH values for 120 h, showing strong acid/alkali corrosion resistance.^[^ ^54b^ ^]^ Xue et al. also confirmed that the superhydrophobic P(VDF‐HFP)/SiO_2_ film was highly stable against chemical corrosion, and could keep almost unchanged cooling capacity even after immersion in strong acid (PH = 1) and alkali solutions (PH = 13).^[^ ^25a^ ^]^

Based on the above design strategies and fabrication techniques already discussed, we selected some representative works with excellent environmental durability listed in **Table** **4** as a reference for readers.

**Table 4:** Selected works showing excellent anti‐environmental aging performance of PDRC materials.

Materials	Fabrications	*R̅*_solar_/${\bar{\varepsilon }}_{8-13\mathrm{um}\;}$	***θ*** ^*^/ *θ*_roll_	Anti‐soiling test	UV test	Thermal test	Anti‐corrosion test	Real‐world test	Ref.
Porous PVdF‐HFP film	Phase‐inversion	0.96/0.97	110°/NA	Dye liquid	NA	14 days (80 °C)	NA	1 month	[19a]
Porous PMMA film	Template method	0.95/0.95	156°/NA	NA	480 h (0.71 W m^−2^)	NA	NA	40 days	[19c]
PVdF/PDMS film	Liquid templating method	0.965/0.943	163.5±1.2°/2.0± 0.6°	Muddy water	NA	NA	Immersion in acidic or alkali solutions for 1 month	1 month	[22c]
Poly (TMPTA‐VTES) coating	UV‐curing method	0.96/0.98	158±2°/4°	NA	50 days (≈2.0 mW cm^−2^)	20 days (80 °C)	Immersed in hydrochloric acid solution or potassium hydroxide solution for 7 days	NA	[25b]
Porous cellulose aerogel	Freezing‐shaping method	0.93/0.91	152°/5°	Dust (brick slag)	NA	NA	NA	NA	[78]
Porous PDMS film	Emulsion templating technology	0.951/0.931	165.7°/2.1°	Sand	10 days (300 W)	NA	Immersion in solutions with different pH values from 1 to 14 for 7days	NA	[88]
Cellulose nanofibers‐based wood	Bulk process	0.96/>0.90	≈150°/NA	NA	NA	NA	NA	NA	[16a]
Melamine‐formaldehyde bulk	Pressing method	0.94/0.95	NA	NA	NA	Flame burning for 15 s	Immersion in solutions of pH = 1, pH = 13 for 24 h	NA	[24]
Poly (Lactic Acid)‐based aerogel	Thermally induced phase separation	0.89/0.93	152°/NA	Potassium permanganate	NA	NA	NA	NA	[17c]
P(VDF‐HFP)/SiO_2_ composite film	Phase separation technology	>0.93/>0.97	163±1°/0.1± 0.1°	Dyed sands	200 h (300 W)	NA	Immersion in acid or alkali solution for 72 h	NA	[25a]
Porous TiO_2_ nanoparticles packed coating	Evaporation‐driven assembly	0.93/0.97	158°/2°	ASTM D7897‐18	1000 h (0.89 W m^−2^)	1000 h (100 °C)	NA	Over 6 months	[18c]
Mica and TiO_2_ particles layer/particles embedded in TPX matrix/silver‐aluminum sheet trilayer film	Particles coating method	>0.9/>0.8	159°/2°	Dye solution (Methyl red, Methyl blue, and Methyl orange)	5 h (0.68 W m^−2^)	NA	NA	NA	[80a]
Micropattern‐based PTFE layer /ceramic porous layer bilayer film	Plasma‐induced thermal‐field assisted oxidation and cross‐linking method	0.88/>0.92	159.7±2.5°/6.8°	Ink, dust and Fe_3_O_4_ powder	NA	100 cycles (400 °C).	Chemical damage	NA	[52b]
SiO_2_ microspheres/silicon oxynitride film/Ag reflective layer trilayer film	Vacuµm technologies and a modified Langmuir–Schaefer self‐assembly process	0.964/0.946	≈130°/NA	NA	720 h (two 40 W UV lamps)	NA	Immersion in water for 3 weeks	NA	[23]
Pigments and SiO_2_ particles embedded in resin layer/silver‐aluminium alloy sheet bilayer film	Roller coating and spray coating	>0.90/>0.85	160°/NA	Rapeseed oil, alcohol solution, dust	100 h	NA	NA	NA	[35a]
Colorful ZrO_2_‐based bilayer coating	Spraying method	>0.95/>0.85	>150°/NA	Dust	120 h	240 h (80 °C)	NA	NA	[52a]
Micropyramid‐arrayed polymer matrix with random ceramic particles	Microstamping method	0.95/>0.96	≈138°/NA	Dye liquid	NA	NA	NA	NA	[80b]
Composite polymer film doping K_2_Ti_6_O_13_) nanofibers	Roll‐to‐roll electrospinning method	0.94/0.91	NA	NA	720 h under sunlight	NA	NA	30 days	[22b]
P(VDF‐HFP) fiber frameworks adhered to SiO_2_ nanoparticles	Electrospinning and electrospraying	0.985/0.95	156°/2.2°	Dust	10 days (300 W)	NA	Immersion in various pH solutions for 5 days	NA	[79]
PTFE particles‐embedded cellulose fiber film	Air‐spraying	0.93/>0.90	165°/NA	Black dye and garden soil	30 days (3 mW cm^−2^)	30 days (−20, 60, and 80 °C, respectively)	Immersion in water for 15 mins	NA	[86b]
SiO_2_ metafibers‐based film	Electrospinning	0.97/>0.90	156°/NA	Mud, soil	30 days (≈60 Mw cm^−2^, 6.5 h per day)	burning at 1256 °C for 300 s	Immersion in acid solution for a week	2 months	[51]
Hierarchically patterned nanoporous film	Template‐molding method	0.921/	158±2°/NA	Dust particles, Gram‐negative Escherichia coli	NA	NA	NA	1 month	[77]
Porous PES – TiO_2_ nanoparticles – Al_2_O_3_ nanoparticles trilayer film	Spray‐coating	0.97/0.92	168°/NA	NA	1000 h (40 W)	NA	NA	7 days	[42]

### 4.4 Challenges and Opportunities

While many successful air voids‐based, pigments‐based, and micro‐patterned strategies have been developed for building AEA‐PDRC, challenges and issues still remain. The major technical ones in our view are listed below, as well as corresponding potential solutions:

1. Optimizing optical and wettable performance with minimal use of material. Pigments, as one of the oldest and classical strategy to construct AEA‐PDRC, possess ideal surface micro‐nano structures to achieve super‐liquid‐repellency. In addition, the inherent environmental aging resistance of inorganic pigments makes the derived materials resistant to photothermal aging, chemical corrosion and so on. However, challenges such as the narrow band gap width of inorganic pigments lead to light absorption in specific bands, thus limiting the maximum theoretical limit of *R̅*_solar_. Typically, the high *R̅*_solar_ of pigments‐based AEA‐PDRC materials is accomplished by substantially increasing the amount of pigments filler, however this is not an optimal solution for optical design. Although air voids‐based strategies have brought the optical properties of PDRC materials to the state‐of‐the‐art level, the surface roughness shaped by the porous structure has yet to be improved. As of now, the surfaces of air‐void‐based AEA‐PDRC materials remain relatively flat, exhibiting insufficient micro‐ and nano‐roughness, which translates into a sizable droplet roll‐off angle, directly impacting their self‐cleaning performance. Lastly, there is still room for further improvement in the large‐scale fabrication and application of micro‐patterned AEA‐PDRC materials. In the future, synergies between multiple strategies should be worth exploring.
2. Enhancing surface robustness. It's essential to recognize that only a small portion of the total surface area of super‐repellent surfaces is in contact with liquids, which places localized high pressure on the surface microstructures under mechanical loads.^[^ ^62b^ ^]^ This inherent vulnerability renders these surfaces prone to fragility, abrasion, and even failure, posing a significant challenge for superhydrophobic PDRC materials, regardless of whether they are constructed using pigments or air‐voids. One potential approach to address this challenge is to bolster the adhesion between the coating and the substrate by introducing adhesives, particularly effective for matrix‐free coatings with delicate micro‐nano structures. However, this self‐sacrificing‐based strategy may not fully resolve the robustness of the top layer and could potentially compromise optical performance over extended periods of use, given the trade‐off with coating thickness. An alternative strategy involves decoupling surface wettability and mechanical stability by optimizing the design at each structural scale. For instance, creating a resilient “micro‐armor” that can withstand wear may offer a solution, although its potential impact on optical properties warrants further exploration.
3. Strengthening environmental durability. Choosing raw materials with stable chemical bonds is usually a common strategy to enhance environmental durability. Nevertheless, environmental durability is a multifaceted challenge, encompassing factors such as photothermal resistance, resistance to chemical corrosion, mechanical stability, and more. Notably, different aspects of durability can at times be mutually exclusive. For instance, while the assembly structure of inorganic pigments offers superior photothermal durability, it may compromise mechanical stability due to the absence of polymer binders. Therefore, exploring integrated strategies that balance and guarantee different durability should gain more attention in the future.
4. Reducing glare and improving colorism. The potential for specular reflection or the manifestation of a singular, blindingly white appearance due to the high light scattering of PDRC materials can lead to issues characterized by excessive brightness disparities and a lack of color diversity.^[^ ^20b^ ^]^ One viable avenue for improvement involves the incorporation of a top colorant layer. A common approach entails the addition of a pigments‐based colorant layer, which serves a dual purpose. Not only does it effectively diminish glare and enrich coloration, but it also fosters the development of nanostructures that resist contamination. However, environmental concerns associated with pigments‐based approaches warrant attention. An alternative strategy revolves around the creation of a structural color layer founded on natural substances like cellulose. This approach holds the promise of circumventing the introduction of inorganic contaminants. Remarkably, there is a dearth of reported studies pertaining to the establishment of super‐repellent PDRC materials rooted in cellulose, highlighting a promising avenue for future research and innovation in this domain.
5. Seeking environmentally friendly raw materials and process routes. Currently, the raw materials utilized in the preparation of AEA‐PDRC predominantly include non‐degradable inorganic pigments, polymers, and environmentally harmful fluorine compounds, owing to the demand for photothermal durability and the requisite surface modifications. Moreover, the fabrication process for AEA‐PDRC materials typically relies on organic solvents such as acetone, toluene, ethanol, and ether as carriers and diluents. Regrettably, the evaporation of these solvents inevitably generates volatile organic compounds, resulting in adverse consequences for atmospheric quality and human health.^[^ ^15^, ^43^ ^]^ In the future, more attention should be paid to the development of natural components‐aqueous solvent‐based AEA‐PDRC materials.

## 5 Conclusions and Outlook

In this review, we provide an overall summary of recent advances in the field of anti‐environmental aging PDRC (AEA‐PDRC). This interdisciplinary research category requires a clever exploitation of commonalities related to different types of durability in order to facilitate the development of comprehensive anti‐aging performances. We specifically focus on the optimization of optical properties and raw material selection for PDRC, as well as the general design principles of contamination resistance, including the construction of liquid repellent surfaces and photocatalysis‐induced superhydrophilic surface. We also examine the internal damage causes of PDRC resulting from UV exposure, thermal aging, flame burning and chemical corrosion, and corresponding characterization methods. Lastly, the designs, fabrication techniques, and performances of PDRC materials that can maintain long‐term working in real‐world environment are reviewed and summarized.

First of all, the development of new strategies and materials for establishing AEA‐PDRC with comprehensive environmental‐aging resistance is of utmost importance. Outdoor PDRC materials are primarily affected by environmental pollution, wind erosion, UV radiation and thermal aging. Therefore, it is critical to prioritize resistance to soiling deposition, photothermal aging, chemical corrosion, and rain impact, while other durability's can be considered secondary. Among them, UV aging resistance and contamination resistance are two of the most significant and challenging to design. UV resistance is typically accomplished by employing organic materials with stable chemical bonds or by directly using inorganic compounds and removing fragile polymeric components. To achieve contamination resistance, a popular strategy is to create a liquid repellent self‐cleaning surface based on optical scatterers supplemented by appropriate structural design and chemical modification. This strategy is widely applicable, regardless of whether it is based on air‐voids, pigments, or photonics. While most of these strategies can improve durability to some extent when utilized alone, the effects of diversified synergistic strategies still require further exploration. For example, the delicate surface texture and unstable air cushion present a major obstacle and challenge to the liquid repellent surface. However, most strategies to improve robustness and durability of liquid repellency have not yet to be applied to AEA‐PDRC. Thus, developing new strategies and advanced composites that can substantially balance and guarantee multiple anti‐aging performances to build comprehensively AEA‐PDRC materials should be a priority for future research.

Second, standard and universal assessments should be established to evaluate the aging resistance of PDRC materials. Currently, there is a lack of uniformity in anti‐aging tests of PDRC materials, which impedes the comparison of results across different studies. For instance, parameters of soiling, such as surface energy, size, and appearance, directly affect the self‐cleaning performance of liquid‐repellent surfaces. Additionally, the intensity and exposure time of UV irradiation have varying effects on PDRC materials. Therefore, it is significant to determine which accelerated simulation test best mimics real‐world environmental aging conditions. To address these issues, it is recommended that ASTM standards and other industrial standards should be utilized for their reliability and repeatability. The use of these unified standard tests can greatly improve our understanding of environmental aging mechanisms, thereby promoting the development and application of PDRC toward practicality.

Last but not least, it is still meaningful to develop simple, low‐cost and green routes/raw materials to build environmentally friendly AEA‐PDRC materials. For example, traditional solvent‐based conversion methods, commonly‐used to build porous structures, rely on irritant and toxic organic solvents like acetone and ethanol, restricting their large‐scale application. Therefore, alternative approaches utilizing greener solvents are being explored. Furthermore, the use of non‐degradable inorganic pigments such as TiO_2_ and BaSO_4_ needs to be minimized, if not eliminated, as they pose a threat to the environment. Nano‐cellulose technology that allows achieving the same or even better optical performances of commercial scattering enhancers is a promising research direction. Another critical concern is the use of fluoride compounds, commonly‐employed for surface modification, which lead to serious health risks. These compounds may face partial or complete prohibition in the near future.

## Conflict of Interest

The authors declare no conflict of interest.
